# Vertical effects of cervical headgear in growing patients with Class II malocclusion: a systematic review and meta-analysis

**DOI:** 10.1093/ejo/cjad053

**Published:** 2023-10-22

**Authors:** Umar Hussain, Ahsan Memood Shah, Fazli Rabi, Alessandra Campobasso, Spyridon N Papageorgiou

**Affiliations:** Department of Orthodontics, Saidu College of Dentistry, Khyber Pakhtunkhwa, Swat, Pakistan; Department Orthodontics, Khyber College of Dentistry, Peshawar, Khyber Pakhtunkhwa, Pakistan; Department of Orthodontics, Saidu College of Dentistry, Swat, Khyber Pakhtunkhwa, Pakistan; Department of Clinical and Experimental Medicine, University of Foggia, Via Rovelli 50, 71122 Foggia, Italy; Clinic of Orthodontics and Pediatric Dentistry, Center of Dental Medicine, University of Zurich, Plattenstrasse 11, 8032 Zurich, Switzerland

**Keywords:** orthodontics, Class II malocclusion, cervical pull headgear, clinical trials, systematic review, meta-analysis

## Abstract

**Background:**

Cervical headgear (cHG) has been shown to be effective in Class II correction both with dental and orthopaedic effects but has traditionally been associated with vertical adverse effects in terms of posterior mandibular rotation.

**Objective:**

To assess the treatment effects of cHG treatment in the vertical dimension.

**Search methods:**

Unrestricted literature search of five databases up to May 2023.

**Selection criteria:**

Randomized/non-randomized clinical studies comparing cHG to untreated controls, high-pull headgear (hp-HG), cHG adjuncts, or other Class II treatment alternatives (functional appliances or distalisers).

**Data collection and analysis:**

After duplicate study selection, data extraction, and risk-of-bias assessment according to Cochrane, random-effects meta-analyses of mean differences (MD)/standardized mean diffences (SMD) and their 95% confidence intervals (CIs) were performed, followed by meta-regressions, sensitivity analyses, and assessment of certainty on existed evidence.

**Results:**

Two randomized/16 non-randomized studies (12 retrospective/4 prospective) involving 1094 patients (mean age 10.9 years and 46% male) were included. Compared to natural growth, cHG treatment was not associated on average with increases in mandibular (eight studies; SMD 0.22; 95% CI −0.06, 0.49; *P* = 0.11) or maxillary plane angle (seven studies; SMD 0.81; 95% CI −0.34, 1.95; P=0.14). Observed changes translate to MDs of 0.48° (95% CI −0.13, 1.07°) and 1.22° (95% CI −0.51, 2.94°) in the SN-ML and SN-NL angles, respectively. No significant differences were seen in *y*-axis, facial axis angle, or posterior face height (*P* > 0.05). Similarly, no significant differences were found between cHG treatment and (i) addition of a lower utility arch, (ii) hp-HG treatment, and (iii) removable functional appliance treatment (*P* > 0.05 for all). Meta-regressions of patient age, sex, or duration and sensitivity analyses showed relative robustness, while our confidence in these estimates was low to very low due to the risk of bias, inconsistency, and imprecision.

**Conclusions:**

cHG on average is not consistently associated with posterior rotation of the jaws or a consistent increase in vertical facial dimensions among Class II patients.

**Registration:**

PROSPERO registration (CRD42022374603).

## Introduction

### Rationale

Since its introduction more than a century ago, the use of extraoral traction with headgear (HG) has gained a prominent place in orthodontic therapy for a wide spectrum of dental goals (including exerting influence on the sagittal or vertical position of the upper molars and expanding the dental arch), orthopaedic goals (retardation of maxillary growth) [1, 2] or as a means to reinforce orthodontic anchorage.

Extraoral traction with HG is usually categorized according to the direction of the applied force into cervical headgear (cHG), high-pull headgear (hp-HG), or combination (both low- and high-pull) HG. Several authors have reported differential treatment effects according to the direction of the applied force from HG: (i) cHG tends to extrude the maxillary dentition, rotate more the mandible backward more than hp-HG [1, 3, 4], and might lead to open-bite [5]; (ii) hp-HG leads to greater forward movement of the chin than cHG but might not be as effective as the latter for severe protrusion cases [3, 6, 7].

Furthermore, several authors have cautioned in the past against the use of cHG especially in dolichofacial patients, as it might lead to molar extrusion, which in turn induces clockwise (backward) mandibular rotation and an increase in mandibular plane angle, thereby worsening a potentially already unattractive profile [1, 4, 8–10].

On the other hand, such backward rotational effects have been reported to be reversible, so that anterior growth of both jaws is eventually seen and other factors such as occlusal forces or occlusal contacts might also influence the final outcome of cHG treatment [9]. Moreover, other authors have reported that cHG did not cause more molar eruption than would be expected from normal eruption [11] and did not produce excessive backward rotation of the mandible [12, 13] even for dolichofacial patients [11], while the vertical skeletal relationships in the growing face could not be predictably altered by cHG treatment [14]. Finally, even if adverse effects on the vertical dimension can be expected from cHG treatment, some adjuncts like incorporation of a lower utility arch from Rickett’s bioprogressive therapy have been suggested to minimize these [15].

A previous systematic review on the subject [10] assessed treatment effects solely from groups of patients treated with cHG and was limited to descriptive analysis without any proper quantitative synthesis (meta-analysis) of the relative effects compared to normal growth, hp-HG, or other Class II treatment alternatives, thereby failing to draw definite conclusions.

### Objectives

The aim of this systematic review was to assess clinical evidence on the vertical effects of cHG in growing skeletal Class II patients. The review aimed to answer the following focussed question: Does treatment with cHG for growing patients with Class II malocclusion have an effect on vertical cephalometric measurements of the craniofacial complex other than could be expected from natural growth? Secondarily, the present review aimed to compare the effects of cHG to the use of any other treatment adjuncts (like utility arches), hp-HG, or other treatment alternatives for Class II treatment (like functional appliances or molar distalization appliances).

## Materials and methods

### Registration and protocol

This review was conducted according to the Cochrane handbook [16] and reported according to the Preferred Reporting Items for Systematic Reviews and Meta-Analyses (PRISMA) 2020 statement [17]. Its protocol was developed a priori following the corresponding PRISMA extension [18], pre-registered (CRD42022374603), and all *post hoc* changes to the protocol were transparently reported (Supplement).

### Eligibility criteria

The eligibility criteria were developed based on the PICOS (participants, intervention, comparison, outcomes, and study design) principle; P: growing patients of any sex with Class II malocclusion without any craniofacial syndrome or anomalies; I: cHG alone or in combination with fixed appliances; C: no treatment (observation of natural growth), cHG with any adjuncts, hp-HG, or any other intraoral appliance; O: vertical cephalometric measurements; and S: clinical comparative studies, including randomized trials and prospective/retrospective cohort (before-and-after) studies. Excluded were case series (defined as studies with <10 patients), case reports, animal, and non-clinical studies. The review’s primary outcome was the inclination of the mandibular plane assessed with the sela-nasion mandibular plane (SN-ML) or the Frankfort horizontal mandibular plane (FH-ML) angle. Secondary outcomes included (i) the inclination of the maxillary plane assessed with the sela-nasion maxillary plane (SN-NL) or the Frankfort horizontal maxillary plane (FH-NL) angle, (ii) the *y*-axis to the anterior cranial base (N-S-Gn), (iii) the facial axis angle (BaN-PtGn), (iv) the lower posterior face height (Ar-Go), and the (v) total posterior face height (S-Go).

### Information sources and search strategy

An unrestricted literature search of five electronic databases (Medline via PubMed, Scopus, Web of Science, Cochrane CENTRAL, and LILACS) was conducted from inception up to 1 May 2023, using an appropriate search strategy (Supplementary Table 1). No restrictions regarding publication date, language, type, or status were used, while the reference lists of eligible articles or previous systematic reviews were manually searched for any additional relevant articles.

### Selection process

Initially, the titles and/or abstracts of all studies identified by the literature search were assessed against the eligibility criteria, followed by retrieval and assessment of their full texts. Study selection was performed independently by two authors (UM and FR) and any discrepancies were resolved by discussion with a third author (AMS).

### Data collection process and items

Data collection was performed using pre-defined and piloted extraction forms covering (i) study characteristics (design, clinical setting, and country); (ii) patient characteristics (age and sex); (iii) appliance characteristics; (iv) measured outcomes; and (v) follow-up duration. To ensure accuracy and consistency, all data was extracted independently by two authors (UH and FR), while any discrepancies were again resolved by discussion with a third author (AMS).

### Study risk of bias

The risk of bias of randomized trials was assessed with the Cochrane risk of bias in randomized trials (RoB 2) tool [19]. The risk of bias within included non-randomized studies was assessed with the ROBINS-I (risk of bias in non-randomized studies of interventions) tool [20]. The risk of bias was assessed independently by two authors (UM and FR) and any discrepancies were resolved by consulting another author (AC).

### Effect measures and synthesis measures

Studies were considered eligible for pooling if similar participants, interventions, and comparisons existed, and sufficient data was reported. In case of missing/partial data provided, we tried to calculate the missing data ourselves (Supplement). The mean difference (MD) with its 95% confidence interval (CI) was generally chosen as effect measure, while the standardized mean difference (SMD) was used to combine cephalometric variables measuring similar outcomes (like SN-ML and FH-ML). As the effects of cHG were expected to vary according to the patient’s chronological/skeletal age, sex, baseline skeletal configuration, angulation of the HG’s inner/outer arms, and patient compliance, a random-effects model was a priori deemed more appropriate to calculate the average distribution of cHG effects across the various scenarios, based on clinical and statistical reasoning [21]. A restricted maximum likelihood variance estimator was chosen, and the CIs were adjusted with the Hartung–Knapp–Sidik–Jonkman method [22, 23]. Between-study heterogeneity was assessed through inspection of forest plots, the tau^2^ (absolute heterogeneity) or the *I*^2^ statistic (relative heterogeneity; inconsistency) and uncertainty intervals were calculated around them [24]. Heterogeneity was assessed in absolute/relative terms, based on its localization on the forest plot, its effect on the summary estimate, and uncertainty around them. To appropriately interpret the results of the random-effects model, 95% predictions were calculated to incorporate existing heterogeneity and provide a range of possible effects for a future clinical scenario [25]. Contour-enhanced forest plots [26] were constructed to visualize the magnitude of observed effects (Supplement) and assess heterogeneity, clinical relevance, and imprecision.

*Post-hoc* random-effects meta-regressions were performed for meta-analyses with at least five studies to assess the impact of patient age, % of male patients, and follow-up duration on the treatment results. Sensitivity analyses were performed again for meta-analyses with at least five studies to assess the impact of (i) study design (randomized or non-randomized studies), (ii) timing of data acquisition (prospective or retrospective studies), (iii) sample size (up to or more than 50 patients per study; arbitrarily chosen), and (iv) our certainty on the meta-analytical estimates (quality of clinical recommendations).

Statistical analyses were run in R statistical software (version 4.0.4; R Foundation for Statistical Computing, Vienna, Austria) by one author (SNP) with an openly provided dataset [27]. All *P*-values are two‐sided with alpha = 0.05, except for tests of between‐studies or between‐subgroups heterogeneity where alpha was set at 0.10.

### Reporting bias assessment and certainty assessment

Reporting biases (including small-study effects and the possibility of publication bias) were planned to be assessed for meta-analysis of at least 10 studies, but no such meta-analyses could ultimately be done.

Our certainty around the meta-analysis results was assessed with the grades of recommendations, assessment, development, and evaluation (GRADE) approach [28] and summarized with revised summary of findings tables [29].

## Results

### Study selection

**Figure 1.**
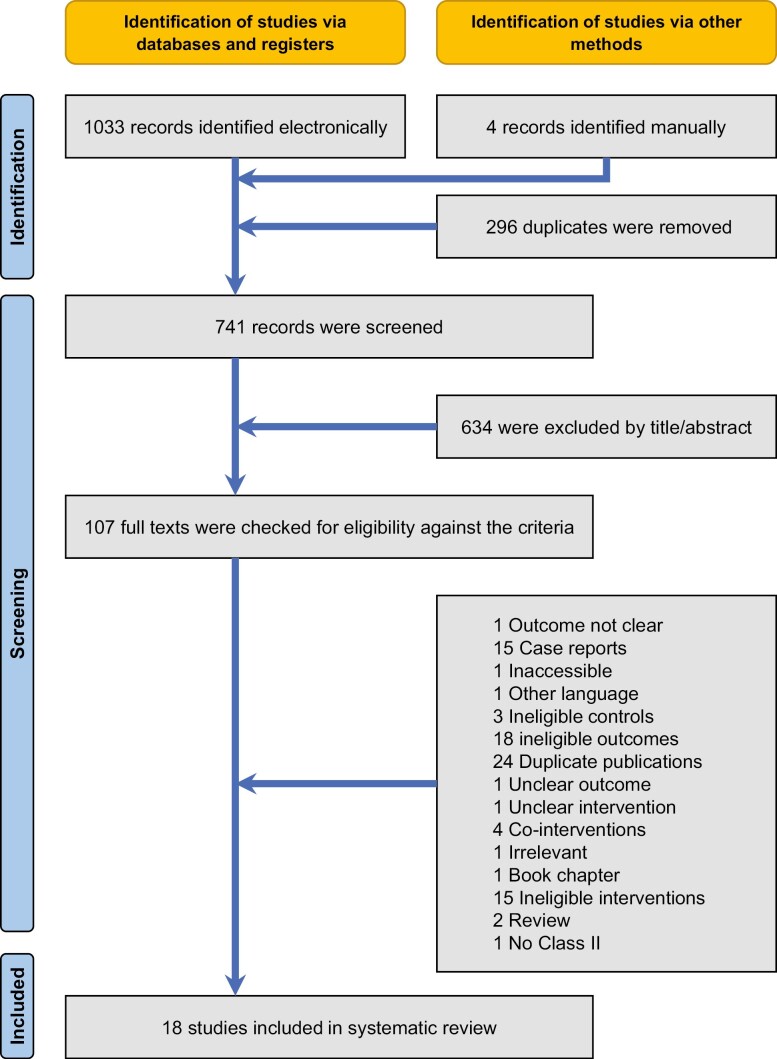
PRISMA flow diagram for the identification and selection of studies eligible for this review.

The electronic database search yielded a total of 1033 records, while another four were identified manually (Fig. 1). After removal of 296 duplicates, 741 records remained for further evaluation and were checked against the eligibility criteria (Supplementary Table 2). Four publications from the same research team from Italy were identified [12, 30–32] and after communication with the authors were grouped as a single clinical study with multiple comparisons. In the end, 21 publications pertaining to 18 unique clinical studies were included in the quantitative and qualitative synthesis.

### Study characteristics

The characteristics of the 18 included studies are shown in Table 1. The majority of studies (67%; 12/18) were retrospective non-randomized cohort (before-and-after) studies, 22% (4/18) were prospective non-randomized cohort (before-and-after) studies, and 11% (2/18) were randomized clinical trials of parallel design. Included studies were conducted in university clinics or private practices in 11 different countries (Brazil, Denmark, Finland, Greece, Italy, Republic of Korea, Spain, Sweden, Switzerland, Turkey, and USA). These 18 studies included a total of 1094 patients (median 57 patients/study and 26 patients/study group), who were on average 45.7% male (423/925; from the 15 studies reporting on sex) and on average 10.9 years old (from the 17 studies reporting on age). Among the 18 included studies, more than half of them (56%; 10/18) compared cHG to an untreated control group, three of them (17%) to a cHG group with a lower utility arch, five of them (26%) to a hp-HG group, four of them (21%) to an intraoral distaliser group, and two of them (11%) to a functional appliance group. Most studies included patients of any vertical skeletal configuration, while four studies (22%) included only hyperdivergent patients and another two studies (11%) included normo- or hyperdivergent patients. The median treatment duration (and study follow-up) was 24.3 months.

**Table 1.** Characteristics of included studies.

Nr	Study	Design; setting^*^	Groups	Patients (M/F); age^†^	Selected patients by vertical classification	Duration (months)
1	Aliò-Sanz (2012)	pNRS; ESP	E: cHG C: No Tx	E: 41 (20/21); NR C: 38 (22/16); NR	Any	42.0
2	Antonarakis (2014)	rNRS; CHE	E: cHG (+FA_BOTH_) C: hp-HG (+FA_BOTH_)	E: 30 (15/15); 10.8 C: 30 (15/15); 10.8	Any	46.8
3	Bondermark (2005)	RCT; SWE	E: cHG C: Intraoral distaliser	E: 20 (8/12); 11.5 C: 20 (10/10); 11.4	Any	6.4
4	Burke (1992)	rNRS; USA	E: cHG (+/−FA_BOTH_) C: hp-HG (+/−FA_BOTH_)	E: 21 (NR); 10.2 C: 32 (NR); 10.5	Hyperdivergent (SN-ML > 34º)	43.3
5	Cook (1994)	rNRS; USA	E1: cHG E2: cHG + LUA C: No Tx	E1: 30 (14/16); 8.6 E2: 30 (21/9); 8.7 C: 30 (15/15); 9.1	Any	19.0
6	Derringer (1990)	rNRS; DNK	E: cHG C1: Activator C2: No Tx	E: 40 (20/20); 11.7 C1: 30 (15/15); 11.8 C2: 22 (11/11); 11.7	Any	51.1
7	Freitas (2008)	rNRS; BRA	E: cHG (+FA_MND_) C: No Tx	E: 25 (5/20); 10.4 C: 16 (4/12); 9.9	Any	30.0
8	Gkantidis (2011)	rNRS; GRC	E: cHG (+FA_BOTH_) C: hp-HG (+FA_BOTH_ + 4 PM-Ex)	E: 28 (14/14); 11.0 C: 29 (13/16); 11.8	Hyperdivergent (SN-ML > 32º)	28.8
9	Haralabakis (2003)	rNRS; GRC	E: cHG C: Activator	E: 30 (9/21); 10.9 C: 22 (11/11); 10.2	Any	27.5
10	Kim (2000)	rNRS; USA	E: cHG (+FA_BOTH_) C: No Tx	E: 30 (7/23); 11.1 C: 26 (10/16); 11.1	Any	49.1
11	Lione (2014)	rNRS; ITA	E: cHG (+FA_BOTH_) C1: Intraoral distaliser (+FA_BOTH_) C2: No Tx	E: 40 (15/25); 11.5 C1: 40 (19/21); 11.6 C2: 25 (12/13); 11.4	Any	18.0
12	Mantysaari (2004)	RCT; FIN	E: cHG C: No Tx	E/C: 68 (40/28); 7.6	Any	16.0
13	Mossaz (2005)	pNRS; CHE	E: cHG (+FA_BOTH_) C: Intraoral distaliser (+FA_BOTH_)	E: 30 (NR); 11.7 C: 30 (NR); 11.6	Normo-/hyperdivergent (NL-ML 20-35)	24.5
14	Park (2017)	rNRS; KOR	E: cHG C: Intraoral distaliser (skeletal)	E: 22 (6/16); 23.0 C: 22 (6/16); 24.7	Any	24.1
15	Rosa (2020)	pNRS; BRA	E: cHG C: No Tx	E: 23 (10/13); 10.7 C: 22 (10/12); 10.7	Any	15.0
16	Sambataro (2017)_collated_	rNRS; ITA	E1: cHG E2: cHG + LUA E3: hp-HG C: No Tx	E1: 20 (10/10); 8.5 E2: 19 (10/9); 8.6 E3: 15 (NR); 9.4 C: 21 (11/10); 8.4	Hyperdivergent (BaN-PtGn < 90º)	21.6
17	Ulger 2006	pNRS; TUR	E1: cHG E2: cHG + LUA C: No Tx	E1: 12 (6/6): 8.9 E2: 12 (5/7); 9.2 C: 12 (4/8); 8.6	Normo-/hyperdivergent	17.2
18	Zervas (2016)	rNRS; USA	E: cHG C: hp-HG	E: 22 (NR); 8.6 C: 19 (NR); 9.4	Hyperdivergent (BaN-PtGn < 90º; TFH > 57º)	10.0

^*^Countries are given with their ISO ALPHA-3 codes.

^†^In years.

cHG, cervical headgear; C, control group; E, experimental group; FA_BOTH_, Fixed appliance on both jaws; FA_MAX_, Fixed appliance on the upper jaw; FA_MND_, Fixed appliance on the lower jaw; hp-HG, high-pull headgear; LUA, lower utility arch; NR, Not reported; PM-Ex; premolar extraction; pNRS, prospective non-randomized study; RCT, Randomised clinical trial; rNRS, retrospective nonrandomised study; TFH, total facial height; Tx, treatment.

### Risk of bias in studies

The risk of bias of the two included randomized trials is given in Supplementary Table 3 and Supplementary Fig. 1. Both randomized trials were judged to be in high risk of bias, due to issues with the randomization process, deviations from intended interventions, and measurement of the outcome of interest. The risk of bias of the 16 included non-randomized studies is given in Supplementary Table 4 and Supplementary Figs. 2 and 3. From these, half of them (50%; 8/16) were judged to be in moderate risk of bias and the other half (50%; 8/16) in high risk of bias. The most problematic domains were bias due to confounding, bias due to the selection of the study’s participants, and bias due to deviations from the intended interventions.

### Results of individual studies and data syntheses

The complete results extracted from all included studies can be found in the review’s openly provided dataset [27]. Results of meta-analyses with at least two studies can be seen in Table 2, while outcomes/comparisons assessed only from single studies can be seen in Supplementary Table 5—the latter not finding any clinically relevant differences between cHG and untreated controls, hp-HG, addition of a lower utility arch to the cHG, intraoral distaliser, functional appliance, or intrusive mechanics.

**Table 2.** Results of meta-analyses (≥2 studies) comparing cervical headgear with other treatment alternatives.

Comparison	Outcome	*n*	Effect (95% CI)	*P*	*τ* ^2^ (95% UI)	*I* ^2^ (95% UI)	95% prediction
cHG vs control	SN-ML/FH-ML	8	SMD 0.22 (−0.06, 0.49)	0.11	0.03 (0, 0.34)	23% (0%, 65%)	−0.31, 0.74
	SN-NL/FH-NL	7	SMD 0.81 (−0.34, 1.95)	0.14	1.11 (0.37, 8.90)	80% (60%, 90%)	−2.11, 3.72
	NSGn	2	MD 1.06 (−6.88, 9.00)	0.34	0.63 (NC)	80% (NC)	NC
	BaN-PtGn	3	MD 0.15 (−0.50, 0.79)	0.43	0 (0, 2.21)	0% (0%, 90%)	−2.52, 2.82
	ArGo	3	MD 2.31 (−2.01, 6.64)	0.15	2.43 (0.25, >100)	82% (43%, 94%)	−21.18, 25.81
	SGo	3	MD 1.30 (−2.99, 5.58)	0.32	2.47 (0.20, >100)	81% (39%, 94%)	−22.65, 25.25
cHG vs hp-HG	SN-ML/FH-ML	4	SMD −0.01 (−0.80, 0.77)	0.96	0.15 (0, 3.29)	63% (0%, 88%)	−2.00, 1.97
	SN-NL/FH-NL	3	SMD 0.51 (−1.15, 2.16)	0.32	0.35 (0.03, 17.17)	79% (34%, 94%)	−8.51, 9.53
	BaN-PtGn	3	MD −0.76 (−4.70, 3.18)	0.49	2.31 (0.48, >100)	92% (80%, 97%)	−23.26, 21,74
cHG vs cHG + lower utility arch	SN-ML/FH-ML	3	SMD 0.08 (−0.47, 0.64)	0.58	0 (0, 1.64)	0% (0%, 90%)	−2.21, 2.38
	SN-NL/FH-NL	3	SMD 0.18 (−0.57, 0.94)	0.41	0 (0, 3.73)	0% (0%, 90%)	−2.14, 2.50
	BaN-PtGn	2	MD −0.33 (−1.59, 0.93)	0.19	0 (NC)	0% (NC)	NC
cHG vs intraoral distaliser	SN-ML	2	MD −0.43 (−3.83, 2.97)	0.36	0.05 (NC)	22% (NC)	NC
	SN-NL/FH-NL	2	SMD 0.02 (−3.26, 3.31)	0.94	0.07 (NC)	48% (NC)	NC
cHG vs functional appliance	SN-ML	2	MD −0.08 (−4.44, 4.28)	0.85	0 (NC)	0% (NC)	NC

cHG, cervical headgear; CI, confidence interval; hp-HG, high-pull headgear; MD, mean difference; NC, not calculable; SMD, standardized mean difference; UI, uncertainty interval.

**Figure 2.**
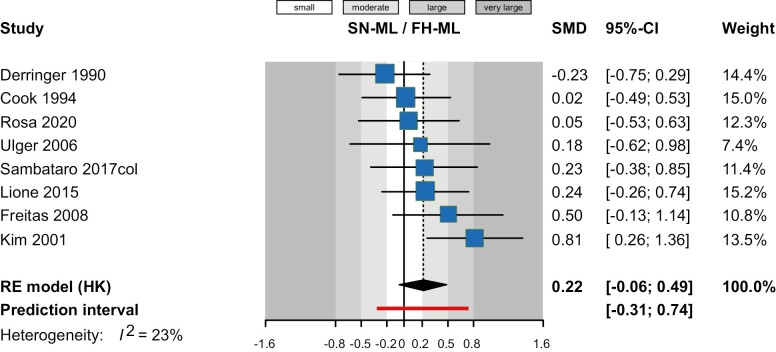
Contour-enhanced forest plot for the effect of cervical headgear versus control (no treatment) on mandibular plane angle (SN-ML/FH-ML). CI, confidence interval; SMD, standardized mean difference.

**Figure 3.**
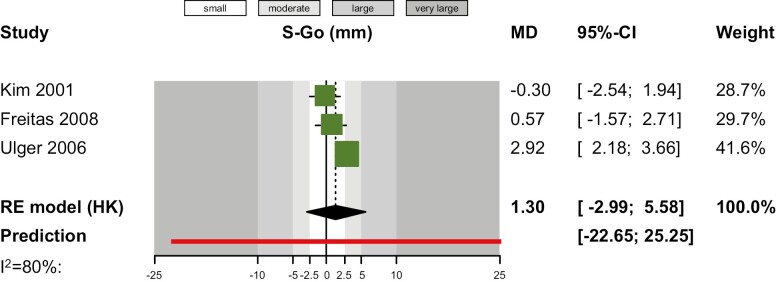
Contour-enhanced forest plot for the effect of cervical headgear versus control (no treatment) on posterior face height (S-Go). CI, confidence interval; MD, mean difference.

As far as comparisons of cHG to natural growth (untreated controls) are concerned, random-effects meta-analyses indicated that cHG was not associated with significantly increased mandibular plane angle (measured with SN-ML or FH-ML) (Table 3). Pooling the results of eight studies (Fig. 2), an SMD of 0.22 was found (95% CI -0.06 to 0.49; *P* = 0.11), which indicated a moderate effect on average and can be back-translated in SN-ML to an increase by 0.48º (95% CI -0.13 to 1.07º.). This is not statistically significant and is less than half the average baseline standard deviation for SN-ML of the control group, which means it is surely of little clinical relevance. Other than that, meta-analyses indicated no differences regarding maxillary plane inclination (through SN-NL or FH-NL; seven studies; *P* = 0.14; Supplementary Fig. 4), *y*-axis (N-S-Gn; two studies; *P* = 0.34; Supplementary Fig. 5), facial axis angle (BaN-PtGn; three studies; *P* = 0.43; Supplementary Fig. 6), lower posterior face height (Ar-Go; three studies; *P* = 0.15; Supplementary Fig. 7), or total posterior face height (S-Go; three studies; *P* = 0.32; Fig. 3).

**Table 3.** Summary of findings table according to the GRADE approach.

	Anticipated absolute effects (95% CI)				
Outcome studies (patients)	Control group^a^	Difference in cHG group	Quality of the evidence (GRADE)^b^	What happens with experimental treatment	Comment
cHG vs control (no Tx)					
** **Mandibular plane inclination (SN-ML) 8 studies (394 patients)	−0.33º	0.48º greater (0.13º lower to 1.07º greater)	⨁◯◯◯ Very low^c^^,^^d^ due to bias, inconsistency	Little to no difference in mandibular plane inclination	Based on an SMD for SN-ML/FH-ML of 0.22 (95% CI -0.06 to 0.49); back-translated to SN-ML using an average control SD of 2.19º.
** **Maxillary plane inclination (SN-NL) 7 studies (391 studies)	+0.16º	1.22º greater (0.51º lower to 2.94º greater)	⨁⨁◯◯ Low^c^^,^^e^ due to bias	Little to no difference in maxillary plane inclination	Based on an SMD for SN-NL/FH-NL of 0.81 (95% CI -0.34 to 1.95); back-translated to SN-ML using an average control SD of 1.51º.
** ** *Y*-axis (N-S-Gn) 2 studies (118 patients)	−0.02º	1.06º greater (6.88º smaller to 9.00º greater)	⨁◯◯◯ Very low^c^^,^^f^ due to bias, imprecision	Little to no difference in *y*-axis	-
** **Facial axis angle (BaN-PtGn) 3 studies (161 patients)	−0.23º	0.15º greater (0.50º smaller to 0.79º greater)	⨁◯◯◯ Very low^c^^,^^f^ due to bias, imprecision	Little to no difference in facial axis	-
** **Posterior face height (S-Go) 3 studies (121 patients)	+3.94 mm	1.30 mm greater (2.99 mm smaller to 5.58 mm greater)	⨁⨁◯◯ Low^c^ due to bias	Little to no difference in posterior face height	-
cHG vs hp-HG					
** **Mandibular plane inclination (SN-ML) 4 studies (189 patients)	+0.83º	0.02º smaller (1.90º smaller to 1.83º greater)	⨁◯◯◯ Very low^c^^,^^f^ due to bias, imprecision	Little to no difference in mandibular plane inclination	Based on an SMD for SN-ML/FH-ML of −0.01 (95% CI -0.80 to 0.77); back-translated to SN-ML using an average control SD of 2.38º.
** **Maxillary plane inclination (SN-NL) 3 studies (136 studies)	0º	1.12º greater (2.53º smaller to 4.75º greater)	⨁◯◯◯ Very low^c^^,^^f^ due to bias, imprecision	Little to no difference in maxillary plane inclination	Based on an SMD for SN-NL/FH-NL of 0.51 (95% CI -1.15 to 2.16); back-translated to SN-ML using an average control SD of 2.20º.
** **Facial axis angle (BaN-PtGn) 3 studies (129 patients)	−0.06º	0.76º smaller (4.70º smaller to 3.18º greater)	⨁◯◯◯ Very low^c^^,^^f^ due to bias, imprecision	Little to no difference in facial axis angle	–

Population: skeletal class II malocclusion; intervention: cervical headgear (+/− braces); comparison: no treatment (control) or high-pull headgear; setting: university clinics or private practices (Brazil, Denmark, Finland, Greece, Italy, Republic of Korea, Spain, Sweden, Switzerland, Turkey, and USA).

^a^Response in the control group is based on the response of included studies (or random-effects meta-analysis of the control response).

^b^Starts from ‘high’.

^c^Downgraded by two levels, due to serious potential issues with confounding, selection of participants, and deviation of intended intervention.

^d^Signs of inconsistency, as potential effects include small reductions to very large increases.

^e^Potential for inconsistency, as the CIs/prediction included a wide range of outcomes. However, this was mostly due to a very heterogeneous study (Ulger 2006) with a very large effect size. Omission of this study led to much more precise estimates (SMD 0.39; 95% CI 0.13 to 0.66; *P* = 0.01). Decided not to downgrade.

^f^Imprecision due to the limited number of small studies.

cHG, cervical headgear; CI, confidence interval; hp-HG, high-pull headgear; SD, standard deviation; SMD, standardized mean difference; Tx, treatment.

Compared to hp-HG (Table 2), treatment with cHG was not associated with increased mandibular plane angle (SN-ML or FH-ML; four studies; *P* = 0.96; Supplementary Fig. 8), maxillary plane angle (SN-NL or FH-NL; three studies; *P* = 0.32; Supplementary Fig. 9), or facial axis angle (BaN-PtGn; three studies; *P* = 0.49; Supplementary Fig. 10).

Addition of a lower utility arch in the cHG protocol had similarly no effect on mandibular plane (SN-ML or FH-ML; three studies; *P* = 0.58; Supplementary Fig. 11) or maxillary plane angle (SN-NL; three studies; *P* = 0.41; Supplementary Fig. 12). Likewise, no significant differences were seen between treatment with cHG and an intraoral distaliser in terms of mandibular (two studies; *P* = 0.36; Supplementary Fig. 13) or maxillary plane angle (two studies; *P* = 0.94; Supplementary Fig. 14). From these two studies, only one was on growing patients and the other was on adult patients (Supplement), but both found no significance difference on maxillary plane inclination (Supplementary Fig. 14)—the only outcome used from the single included study on adult patients. Finally, no significant difference in mandibular plane angle was seen between treatment with cHG and functional appliance (two studies; *P* = 0.85; Supplementary Fig. 15).

Meta-regression analyses found no significant effect of baseline patient age, % of male patients within the study sample, or follow-up duration on the effects of cHG compared to untreated controls—in terms of mandibular or maxillary plane angle (*P* > 0.10 in most instances; Supplementary Table 6). The only exception was patient sex, where male patients were associated with smaller opening of the mandibular plane angle, which was however of very small magnitude.

Finally, sensitivity analyses found no significant differences according to study design (randomized vs non-randomized studies), data acquisition timing (prospective vs prospective studies), or study sample size (up to vs more than 50 patients/study), which indicated robustness of the results (Supplementary Table 7). The only exception was sensitivity analysis by study design, where considerable greater increase in mandibular plane angle was seen in weaker retrospective studies, but not in prospective studies.

### Certainty of evidence

Our certainty on the results of the meta-analysis was assessed with the GRADE approach and was judged as either low or very low in all instances. The greatest issue was the high risk of bias due to the inclusion of non-randomized studies with methodological weaknesses. Furthermore, inconsistency was found on the effect of cHG on mandibular plane angle, as the effect magnitude was unclear and reported effects ranged from small to moderate or large (Fig. 2). Furthermore, signs of inconsistency were seen in the effect of cHG on maxillary plane angle (Supplementary Fig. 4) that was overall not significant (*P* = 0.14) and was very heterogeneous (τ^2^ 1.11; *I*^2^ 80%). This was due to the outlier of the Ülger *et al*. [33] study that showed a much larger effect than all other studies. Omitting this study led to a much more precise meta-analysis that indicated increased maxillary plane inclination with cHG (five studies; SMD 0.39; 95% CI 0.13 to 0.66; *P* = 0.01) that might be more appropriate than the original analysis. This can be back-translated to an increase in SN-NL by 0.59º (95% CI 0.20 to 1.00º), which even though statistically significant, is of little clinical relevance.

## Discussion

### Results in context

The present review systematically appraised evidence from 18 clinical studies and a total 1094 patients being treated with cHG and compared to untreated controls or other Class II treatment alternatives and is to the best of our knowledge the first study of its kind.

The results of the meta-analyses indicated that treatment with cHG was associated with a minimal non-significant posterior rotation of the mandible and the maxilla compared to natural growth (0.48º and 1.22º, respectively), which is however of little clinical relevance. This comes in contrast with a previous narrative analysis of the literature [10] that reported bite opening and increased vertical cephalometric measurements after cHG treatment. This also contradicts previous opinions that cHG is de facto contraindicated for high-angle facial types due to its clockwise (backward) mandibular rotation and increase in mandibular plane angle that can worsen a potentially already unattractive profile [1, 4, 8–10] and hp-HG being a more appropriate choice for such cases [1, 35, 36] Possible explanations for this include among others that the HG-induced molar extrusion is offset by a significant increase in ramus height due to increased condylar growth [11, 12, 33] and therefore no significant increase in mandibular plane angle is seen.

Similarly, the present review failed to find that treatment with cHG resulted in more pronounced backward growth rotation, since no difference in the *y*-axis or the facial axis angle was found with either untreated controls or hp-HG (Table 2). This is in agreement with the notion by Melsen [9] who found that both cHG and hp-HG had a similar effect on the growth direction of the maxilla or mandible and a mostly anteriorly directed mandibular growth is seen after both treatment alternatives.

It has been proposed that cHG is associated with increased height at the maxillary molar that mimics natural growth, produces occlusal interferences, and subsequently leads to forward movement of the mandible to maintain the occlusal contacts [7, 37]. Indeed, this slightly greater extrusion of the maxillary molar seemed to be compensated by a positional stability of the lower molar that extruded significantly less after treatment with cHG than with hp-HG [7] and agrees with previous reports [38].

Combination of cHG with a lower utility arch was not found from the present review to be associated with significantly different vertical effects than treatment cHG alone. Ricketts had propagated that the addition of a utility arch to cHG avoids incisor interference and through this reverse response prevents opening rotation of the mandible [12]. Theoretically, use of a lower utility arch would lead to a stabilizing reverse response for the mandibular plane angle and the facial axis angle [15] or even to a counterclockwise (forward) rotation in patients with strong musculature. However, the utility arch was eventually effective in tipping the lower molar distally and maintaining its mesiodistal position but did not have a significant effect on its vertical position [11, 33].

Class II treatment with cHG was found to have similar effects in the vertical direction with removable functional appliances both in terms of mandibular plane angle (Table 2; Supplementary Fig. 15) and in terms of maxillary plane angle, *y*-axis, and lower posterior face height (Supplementary Table 5). This is in agreement with the observation of Baumrind *et al*. [39] who reported that treatment with cHG was associated with an increase in lower face height compared to natural growth or hp-HG and which was similar to treatment with Activator, but at the same time additional growth at the ramus kept the mandibular plane angle stable. Removable functional appliances are known to induce a small increase in mandibular plane angle, which amount to about 0.66⁰ increase in SN-ML per treatment year compared to natural growth, but no consistent rotational effect on the maxilla [40]. Generally, however, it seems that treatment-related effects from usual Class II correction methods on mandibular plane orientation are too small in themselves to be of major clinical relevance [41, 34] and that cHG treatment simply circumvents the usual reduction in mandibular pane angle of normal growth [42].

Finally, no significant difference in vertical effects was found between cHG and dentally anchored intraoral distalisers. This is logical, as mostly similar extrusive effects for the maxillary molar were seen between the two groups [43, 44].

### Strengths and limitations

This review has several strengths including a priori registration [45], an extensive unrestricted literature search, robust analytical methods [22], sensitivity analyses to check the influence of methodological characteristics on the studies’ results, its transparent open data availability [46], and assessment of our confidence in the meta-analysis results through the GRADE approach.

However, certain limitations exist for this review. First and foremost, most of the included studies were non-randomized, many were retrospective, and some also included historical control groups—study design characteristics that have all been linked to increased risk of bias [47–49]. Furthermore, information like baseline skeletal configuration as selection criterion, the vertical angulation/length of the external HG bows, calculated line of applied force according to the centre of resistance, magnitude of applied forces, and compliance with prescribed wear might influence the observed treatment effects [3, 9, 50–55], but were not adequately reported in included studies and could therefore not be formally assessed statistically in this review that provides the average distribution of cHG effects.

## Conclusions

Based on available evidence from mostly non-randomized clinical studies assessing the effect of Class II treatment with cHG, mostly minor effects on vertical parameters were seen. Compared to natural growth cHG treatment was not consistently associated with increases in the maxillary and mandibular plane angles, while no effects on posterior face height or growth direction were seen. No considerable differences on the vertical effects were seen between cHG and addition of a lower utility arch, hp-HG, functional appliances, or intraoral distalisers. However, our certainty about these findings is limited due to serious methodological limitations of the current evidence base and future studies with more robust design might shed more light on this matter.

## Supplementary Material

cjad053_suppl_Supplementary_Material

## Data Availability

The data underlying this article are openly available at Zenodo: http://doi.org/10.5281/zenodo.8173798 (27).
