# Supplementary material for: Vertical effects of cervical headgear in growing patients with Class II malocclusion: a systematic review and meta-analysis
Source: Eur J Orthod. 2023 Oct 22;46(1):cjad053. doi: 10.1093/ejo/cjad053 (PMC10783157; doi:10.1093/ejo/cjad053)
Supplement: cjad053_suppl_Supplementary_Material [file cjad053_suppl_supplementary_material.pdf]

Vertical effects of cervical cervical headgear in growing patients with Class II malocclusion: a systematic review and meta-analysis

Supplementary Material

Supplement. Additional methods and deviations from protocol.

Methods

- Produced forest plots were augmented with contours denoting the magnitude of observed effects to assess precision, heterogeneity, and clinical relevance. For Mean Differences (MD), effects greater than half, one, and two Standard Deviations (SD) were used as cut-off points to denote small, moderate, large, and very large effects, using the average SD of the control group among studies included in the meta-analyses. For Standardised Mean Differences (SMD), cut-offs of 0.2, 0.5, and 0.8 were used.
- In a couple of instances where medians and interquartile ranges were provided, we took the median to be a close approximation of the mean. The SD was calculated from the interquartile range according to Wan et al. [2014].
- When before-and-after treatment values were provided, these were converted to treatment-induced increments (post minus pre) according to Cochrane guidelines [Higgins et al., 2021] using pre/post correlation back-calculated from data available from other studies.
- The clinical relevance of statistically significant effects was arbitrarily judged as being larger than one standard deviation of the response variable in the control, averaged across eligible studies.

Deviations from protocol

- For the primary scope of the systematic review (cHG versus natural growth from untreated control groups) only growing patients were included. For on of the secondary scopes of the review (cHG versus either cHG adjuncts, hp-HG, intraoral distalisers, or functional appliances) also an identified study who compared cHG versus intraoral distalisers for adult patients [Park et al., 2017] was decided post hoc to be included.

- Initially, analyses of subsets according to methodological characteristics (study design, sample size, etc) were planned in the protocol as subgroup analyses. These are still reported, but as sensitivity analyses, since it was deemed more appropriate.
- Post hoc meta-regressions were attempted according to baseline divergency (according to either SN-ML or FH-ML). However, for each of the two variables, few studies contributed (4-5) and no formal analysis was undertaken.
- Sensitivity analyses comparing randomized and non-randomised studies were ultimately not performed. Only two meta-analyses included enough studies for this sensitivity analysis: (i) one meta-analysis had only non-randomised studies and (ii) the other included one randomized and six non-randomised studies and was deemed unstable for such analysis.
- Reporting biases were originally planned to be conducted (with contour-enhanced funnel plots and Egger's test) for meta-analyses with ≥10 studies [Higgins et al., 2021]. However, all meta-analyses ultimately included less than 10 studies and no such analysis was possible.

References

Higgins, J.P.T, Thomas, J., Chandler, J., Cumpston, M., Li, T., Page, M.J., & Welch, V.A (editors). Cochrane Handbook for Systematic Reviews of Interventions version 6.3 (updated February 2022). Cochrane, 2022. Available from www.training.cochrane.org/handbook.

Wan, X., Wang, W., Liu, J., & Tong, T. (2014). Estimating the sample mean and standard deviation from the sample size, median, range and/or interquartile range. BMC Medical Research Methodology, 14, 135.

Supplementary Table 1. Search strategy in MEDLINE (PubMed) and all other databases for the identification of eligible studies for this review.

((Class II malocclusion OR mandibular deficiency OR maxillary excess OR skeletal malocclusion OR growing patients OR Angle Class II malocclusion) AND (extraoral traction appliance* OR extraoral traction OR Extra-oral traction OR headgear OR cervical headgear OR Cervical pull headgear OR cervical* OR High pull headgear OR high-pull headgear OR facebow OR Face-bow)) AND (Vertical control OR vertical* OR facial height OR face height OR facial divergence OR open bite OR long face OR high angle)

Supplementary Table 2. Studies identified from the literature search with their inclusion / exclusion status (with reasons).

Nr	Title	Status
1	A cephalometric study to compare the effects of cervical traction and Andresen therapy in the treatment of Class II division 1 malocclusi	Excluded; duplicate
2	Activator versus cervical headgear: Superimpositional cephalometric comparison	Excluded; duplicate
3	Cephalometric changes in Class II, Division 1 cases after orthopedic treatment with the bioactivator	Excluded; duplicate
4	Cephalometric changes in growing patients with increased vertical dimension treated with cervical headgear	Excluded; duplicate
5	Cervical headgear treatment and growth patterns: analysis by lateral cephalometry	Excluded; duplicate
6	Class II malocclusion nonextraction treatment with growth control An interview with Mark G. Hans	Excluded; duplicate
7	CONTROL OF THE VERTICAL DIMENSION IN CLASS-II CORRECTION USING A CERVICAL HEADGEAR AND LOWER UTILITY ARCH IN GROWING PATIENTS .1. (VOL 107, PG 376, 1994)	Excluded; duplicate
8	Cook AH, Sellke TA, BeGole EA. Control of the vertical dimension in Class II correction using a cervical headgear and lower utility arch in growing patients. Part I. American Journal of Orthodontics and Dentofacial Orthopedics. 1994 Oct 1;106(4):376-88.	Excluded; duplicate
9	Effects of activator and high-pull headgear combination therapy: skeletal, dentoalveolar, and soft tissue profile changes	Excluded; duplicate
10	Effects of cervical headgear and pendulum appliance on vertical dimension in growing subjects: a retrospective controlled clinical trial	Excluded; duplicate
11	Influence of straight-pull headgear on the eruption pattern of maxillary canines: a retrospective study	Excluded; duplicate
12	Long-term outcome of skeletal Class II Division 1 malocclusion treated with rapid palatal expansion and Kloehn cervical headgear	Excluded; duplicate
13	Mossaz CF, Byloff FK, Kiliaridis S. Cervical headgear vs pendulum appliance for the treatment of moderate skeletal Class II malocclusion. American Journal of Orthodontics and Dentofacial Orthopedics. 2007 Nov 1;132(5):616-23.	Excluded; duplicate
14	Sambataro S, Fastuca R, Oppermann NJ, Lorusso P, Baccetti T, Franchi L, Caprioglio A. Cephalometric changes in growing patients with increased vertical dimension treated with cervical headgear. Journal of Orofacial Orthopedics/Fortschritte der Kieferorthopädie. 2017 Jul;78(4):312-20.	Excluded; duplicate
15	The effect of cervical headgear and lower utility arch on the control of vertical dimension in tooth and jaw]	Excluded; duplicate
16	Treating Class II malocclusion in children. Vertical skeletal effects of high-pull or low-pull headgear during comprehensive orthodontic treatment and retention	Excluded; duplicate
17	Cephalometric variables predicting the long-term success or failure of combined rapid maxillary expansion and facial mask therapy	Excluded by title
18	Craniofacial and airway growth in 9-11 years old normal dental occlusion in Iranian	Excluded by title
19	Dental and skeletal components of Class II open bite treatment with a modified Thurow appliance	Excluded by title
20	Early treatment of class III malocclusion with Petit facemask therap	Excluded by title
21	Initial and late treatment effects of headgear-Herbst appliance with mandibular step-by-step advancement. American journal of orthodontics and dentofacial orthopedics	Excluded by title
22	Long-term effect of the chincap on hard and soft tissues	Excluded by title
23	Long-term follow-up of early treatment with reverse headgear	Excluded by title
24	Orthodontic management of the short face pati	Excluded by title
25	Patterns of change in mandibular and facial shape associated with the use of forces to retract the maxilla	Excluded by title
26	skeletal and dental effects and treatment timing for functional appliances in Class II malocclusion	Excluded by title
27	Skeletal changes of maxillary protraction in patients exhibiting skeletal class III malocclusion: a comparison of three skeletal maturation groups	Excluded by title
28	The orthodontic patient with mixed dentition and sleep disorders	Excluded by title
29	The use of tensor analysis to investigate facial changes in treated Class II division 1 malocclusion	Excluded by title
30	Treatment of Class II deep bite by orthodontic and surgical me	Excluded by title
31	Treatment timing for Twin-block therapy	Excluded by title
32	Vertical changes in high-angle class II	Excluded by title
33	A case of skeletal Class II malocclusion (with a high mandibular plane angle) in the permanent dentition]	Excluded by title
34	A cephalometric comparison of mandibular headgear and chin-cap appliances in orthodontic and orthopaedic view points	Excluded by title
35	A cephalometric study of the class II correction effects of the Eureka Spring	Excluded by title
36	A comparative study of two arbitrary face-bow transfer systems for orthognathic surgery planning	Excluded by title
37	A comparison of hand-wrist bone and cervical vertebral analyses in measuring skeletal maturation	Excluded by title
38	A computed tomographic image study on the thickness of the infrazygomatic crest of the maxilla and its clinical implications for miniscrew insertion	Excluded by title
39	A Correlational Study of Scoliosis and Trunk Balance in Adult Patients with Mandibular Deviation	Excluded by title
40	A cross-sectional retrospective study of normal changes in the pharyngeal airway volume in white children with different skeletal patterns. Part 2: Cervical vertebral maturation method and hyoid bone	Excluded by title
41	A growth-related concept for skeletal class II treatment	Excluded by title
42	A high pull torquing auxiliary for use with the Begg appliance	Excluded by title
43	A longitudinal study of normal asymmetric mandibular growth and its relationship to skeletal maturation	Excluded by title
44	A long-term follow-up study of Class II malocclusion correction after treatment with Class II elastics or fixed functional appliances	Excluded by title
45	A new protocol of Tweed-Merrifield directional force technology with microimplant anchor	Excluded by title
46	A novel approach in treatment of maxillary deficiency by reverse chin cup	Excluded by title
47	A novel approach in treatment of open bite: a case report	Excluded by title
48	A prospective optical surface scanning and cephalometric assessment of the effect of functional appliances on the soft tissues	Excluded by title
49	A prospective optical surface scanning and cephalometric assessment of the effect of functional appliances on the soft tissues	Excluded by title
50	A prospective study of the treatment effects of a removable appliance with palatal crib combined with high-pull chincup therapy in anterior open-bite patients	Excluded by title
51	A retrospective long-term comparison of early RME-facemask versus late Hybrid-Hyrax, alt-RAMEC and miniscrew-supported intraoral elastics in growing Class III patients	Excluded by title
52	A study of cervical vertebra anomalies among individuals with different sagittal and vertical facial growth patterns	Excluded by title
53	A study of highpull extraoral traction on the treatment of growing patients with skeletal Class II malocclusion]	Excluded by title
54	A three miRNAs signature predicts survival in cervical cancer using bioinformatics analysis	Excluded by title
55	Activator headgear therapy	Excluded by title
56	Adult gummy smile correction with temporary skeletal anchorage devices	Excluded by title
57	Age, Sex, and Maxillary Position Are Associated with Successful Microimplant-Assisted Rapid Palatal Expansion in Adults	Excluded by title
58	Aggravation of Gummy Smile by Straight-Wire Mechanics and its Management with or without Orthognathic Surgery Up to 10-Year Follow-Up	Excluded by title
59	AIDS in the Third World: how to stop the HIV infection?	Excluded by title
60	Alternate rapid maxillary expansion and constriction (alt-ramec) may be more effective than rapid maxillary expansion alone for protraction facial mask treatment	Excluded by title
61	Alternative treatment for open bite Class III malocclusion in a child with Williams-Beuren syndrome	Excluded by title
62	An adult case of skeletal Class II with high mandibular plane angle treated by anterior rotation of the mandible with miniscrew anchorage	Excluded by title
63	An appraisal of growth and reaction to extraoral anchorage. Simulation of orthodontic-orthopedic results	Excluded by title
64	An early approach for the interception of skeletal open bite: a preliminary report	Excluded by title
65	An electromyographic evaluation of bilateral symmetry of masticatory, neck and trunk muscles activity in patients wearing a positioner	Excluded by title
66	An evidence-based approach to treatment of open bite and deep bite: case reports	Excluded by title
67	An experimental study of orthopedic therapy in skeletal open bite during growth]	Excluded by title
68	An interview with Mark G. Hans	Excluded by title
69	An interview with: AntÃ´nio Carlos de Oliveira Ruellas	Excluded by title
70	An unusual case of invasive cervical resorption after piezosurgery-assisted en masse retraction	Excluded by title
71	Angle class II correction: stepwise mandibular advancement or bite jumping? A systematic review and meta-analysis of skeletal, dental and condylar effects	Excluded by title
72	Angle Class III malocclusion with anteroposterior and vertical discrepancy in the final stage of growth	Excluded by title
73	Anterior open bite and overjet treated with camouflage therapy	Excluded by title
74	Anterior vertical incremental facial growth: its effects in class II treatment	Excluded by title
75	Anteroposterior and vertical changes in skeletal class II patients treated with modified Thurow appliance	Excluded by title
76	Application and effectiveness of a mini-implant- and tooth-borne rapid palatal expansion device: the hybrid hyrax	Excluded by title
77	Articular compass: the location of frontal accessories of bioelastic appliances	Excluded by title
78	Assessing skeletal relationships using the cervical vertebral curvature	Excluded by title
79	Assessment and Comparison of Cervical Column Morphology and Cranial Base Angle in Three Different Facial Types - A Cephalometric Study	Excluded by title
80	Assessment and Comparison of the Head Posture and Craniofacial Growth in Vertical Dimension-A Cephalometric Study	Excluded by title
81	Assessment of mandibular growth and response to functional appliance treatment in prepubertal patients with different auxologic categories	Excluded by title
82	Assessment of mandibular growth and response to orthopedic treatment with 3-dimensional magnetic resonance images	Excluded by title
83	Assessment of the duration of the pubertal growth spurt in patients with skeletal open bite : AÂ cross-sectional study	Excluded by title
84	Assessment of Upper and Lower Airway Dimensions in Different Growth Patterns in Class I Skeletal Malocclusions	Excluded by title
85	Association of long-term outcome of long cervical fusion with sagittal balance: the significance of T1 slope minus cervical lordosis	Excluded by title
86	Associations among upper airway structure, body position, and obesity in skeletal Class I male patients with obstructive sleep apnea	Excluded by title
87	Banded versus modified appliances for anchorage during maxillary protraction	Excluded by title
88	Calcification of the Atlanto-Occipital Ligament (Ponticulus Posticus) in Orthodontic Patients: A Retrospective Study	Excluded by title
89	Camouflage of a high-angle skeletal Class II open-bite malocclusion in an adult after mini-implant failure during treatment	Excluded by title
90	Camouflage treatment of skeletal Class III malocclusion with multiloop edgewise arch wire and modified Class III elastics by maxillary mini-implant anchorage	Excluded by title
91	Category 5: Class II Division 1 malocclusion	Excluded by title
92	Centrographic analysis of 1-phase versus 2-phase treatment for Class II malocclusion	Excluded by title
93	Cephalometric A point changes during and after maxillary protraction and expansion	Excluded by title
94	Cephalometric and occlusal changes following maxillary expansion and protraction	Excluded by title
95	Cephalometric changes after headgear anchored to the deciduous second molars in the early mixed dentition	Excluded by title
96	Cephalometric changes during headgear-reactivator treatment	Excluded by title
97	Cephalometric effects of combined palatal expansion and facemask therapy on Class III malocclusion	Excluded by title
98	Cephalometric evaluation in different phases of Jasper jumper therapy	Excluded by title
99	Cephalometric evaluation of the effect of dynamax and monoblock appliances on vertical facial height in patients with distal malocclusion]	Excluded by title
100	Cephalometric outcomes of a new orthopaedic appliance for Class III malocclusion treatment	Excluded by title
101	Cephalometric study of Class II Division 1 patients treated with an extendedduration, reinforced, banded Herbst appliance followed by fixed appliances	Excluded by title
102	Cervical and craniocervical posture as predictors of craniofacial growth	Excluded by title
103	Cervical arthroplasty: the beginning, the middle, the end?	Excluded by title
104	Cervical column morphology in patients with skeletal open bite	Excluded by title

105	Cervical headgear effects on the morphology of the cervical vertebrae and cervical posture	Excluded by title
106	Cervical lordosis angle measured on lateral cephalograms; findings in skeletal class II female subjects with and without TMD: a cross sectional study	Excluded by title
107	Cervical retraction of the maxillae in the Macaca mulatta monkey using heavy orthopedic force	Excluded by title
108	Cervical spine curvature and craniofacial morphology in an adult Caucasian group: a multiple regression analysis	Excluded by title
109	Cervical vertebrae anomalies in patients with class III skeletal malocclusion	Excluded by title
110	Cervical vertebrae anomalies in subjects with Class II malocclusion assessed by lateral cephalogram and cone beam computed tomography	Excluded by title
111	Cervical vertebrae maturation, dentoalveolar, head postural and respiratory parameters in predicting the stable outcome of face-mask treatment	Excluded by title
112	Cervical vertebral body fusions in patients with skeletal deep bite	Excluded by title
113	Cervical vertebral column morphology and head posture in preorthodontic patients with anterior open bite	Excluded by title
114	Change of cervical sagittal alignment after the treatment of growing rods to the early-onset scoliosis]	Excluded by title
115	Changes in Cervical Lordosis After Orthognathic Surgery in Skeletal Class III Patients	Excluded by title
116	Changes in cranial base morphology in different malocclusions	Excluded by title
117	Changes in dental arch dimensions in patients with class II, division 1, malocclusion treated with headgear	Excluded by title
118	Changes in dentofacial morphology in skeletal Class III children treated by a modified maxillary protraction headgear and a chin cup: a longitudinal cephalometric appraisal	Excluded by title
119	Changes in facial expressions following functional orthopaedic treatment for Class II division 1 malocclusion: a prospective controlled study	Excluded by title
120	Changes in pharyngeal airway dimensions and hyoid bone position after maxillary protraction with different alternate rapid maxillary expansion and construction protocols: A prospective clinical study	Excluded by title
121	Changes in the craniofacial structures and esthetic perceptions of soft-tissue profile alterations after distalization and Herbst appliance treatment	Excluded by title
122	Changes in the soft tissue profile after extraction orthodontic therapy	Excluded by title
123	Changes of Pharyngeal Airway Size and Hyoid Bone Position Following Orthodontic Treatment of Class II Open Bite Patient	Excluded by title
124	Changes of soft tissue profile in operated unilateral cleft lip and palate patients after maxillary protraction]	Excluded by title
125	Changes of tongue position and oropharynx following treatment with functional appliance	Excluded by title
126	Chincup therapy for a young woman with anterior displacement and obtuse angle of the mandible in Class I malocclusion	Excluded by title
127	Chiropractic/dental cotreatment of lumbosacral pain with temporomandibular joint involvement	Excluded by title
128	Chromatometra or chromatocolpos - technical considerations in the management of obstructed mullerian duct anomalies	Excluded by title
129	Chronic snoring and obstructive sleep apnea-hypopnea syndrome in children]	Excluded by title
130	Class II correction in Herbst and Bass therapy	Excluded by title
131	Class II Division 1 malocclusion with a high mandibular plane angle corrected with 2-phase treatment	Excluded by title
132	Class II Division 1: An Evidence- Based Review of Management and Treatment Timing in the Growing Patient	Excluded by title
133	Class II malocclusion treated with miniscrew anchorage: Comparison with traditional orthodontic mechanics outcomes	Excluded by title
134	Class II malocclusion treatment using high-pull headgear with a splint: a systematic review	Excluded by title
135	Class II treatment effects with fixed functional appliances: Jasper jumper vs. Forsus fatigue resistant device	Excluded by title
136	Class II treatment: Problems and solutions	Excluded by title
137	Class III malocclusion: a comparison of extraction and non-extraction techniques	Excluded by title
138	Class III malocclusion: the post-retention findings following a non-extraction treatment approach	Excluded by title
139	CLASS-III MALOCCLUSION - A COMPARISON OF EXTRACTION AND NON-EXTRACTION TECHNIQUES	Excluded by title
140	Clinical outcomes of Frankel appliance therapy assessed with a counterpart analysis	Excluded by title
141	Combined fixed-functional treatment of skeletal class II malocclusions with the EVAA appliance A preliminary study	Excluded by title
142	Combining traditional techniques to correct anterior open bite and posterior crossbite	Excluded by title
143	Comparative Analysis of Dentoskeletal Changes of the Twin Block Appliance and the AdvanSync2 Appliance in Treatment of Skeletal Class-II Malocclusion in Pakistani Population: A Randomized Clinical Trial	Excluded by title
144	Comparative efficacy of the bone-anchored maxillary protraction protocols for orthopaedic treatment in skeletal Class III malocclusion: A Bayesian network meta-analysis	Excluded by title
145	Comparative evaluation of 2 skeletally anchored maxillary protraction protocols	Excluded by title
146	Comparative study between the SFS and LFS rotation as a possible morphogenic mechanism	Excluded by title
147	Comparative study of the Frankel (FR-2) and bionator appliances in the treatment of Class II malocclusion	Excluded by title
148	Comparison between Classic Twin-block and a Modified Clear Twin-block in Class II, Division 1 Malocclusions: A Randomized Clinical Trial	Excluded by title
149	Comparison of 2 modifications of the twin-block appliance in matched Class Iisamples	Excluded by title
150	COMPARISON OF ANCHORAGE EFFICIENCY OF ORTHODONTIC MINI-IMPLANT AND CONVENTIONAL ANCHORAGE REINFORCEMENT IN PATIENTS REQUIRING MAXIMUM ORTHODONTIC ANCHORAGE: A SYSTEMATIC REVIEW AND META-ANALYSIS	Excluded by title
151	Comparison of direct and indirect methods in retraction of maxillary anterior teeth by mini-screw in orthodontic patients: a randomized clinical trial	Excluded by title
152	Comparison of longitudinal treatment effects with facemask and chincup therapy followed by fixed orthodontic treatment on Class III malocclusion	Excluded by title
153	Comparison of orthodontic treatment outcomes in adults with skeletal open bite between conventional edgewise treatment and implant-anchored orthodontics	Excluded by title
154	Comparison of prospectively and retrospectively selected American Board of Orthodontics cases	Excluded by title
155	Comparison of Protraction Facemask Response Using Banded and Bonded Expansion Appliances as Anchorage	Excluded by title
156	Comparison of the change in inferior sclera exposure after maxillary protraction with or without skeletal anchorage	Excluded by title
157	Comparison of the effects of maxillary protraction using facemask and miniplate anchorage between unilateral and bilateral cleft lip and palate patients	Excluded by title
158	Comparison of the effects of mini-implant and traditional anchorage on patients with maxillary dentoalveolar protrusion	Excluded by title
159	Comparison of the intrusion effects on the maxillary incisors between implant anchorage and J-hook headgear	Excluded by title
160	Comparison of three methods to assess individual skeletal maturity	Excluded by title
161	Comparison of treatment outcomes between skeletal anchorage and extraoralanchorage in adults with maxillary dentoalveolar protrusion	Excluded by title
162	Comparison of two protocols for early treatment of dentoskeletal Class III malocclusion: Modified SEC III versus RME/FM	Excluded by title
163	Complications and outcomes of posterior fusion in children with atlantoaxial instability	Excluded by title
164	Cone beam computed tomography evaluation of the relationship between atlantodental interval and skeletal facial morphology in adolescents	Excluded by title
165	Conservative treatment for a growing patient with a severe, developing skeletal Class III malocclusion and open bite	Excluded by title
166	Control of anchorage in the antero-posterior and vertical planes in edgewise technics]	Excluded by title
167	Control of vertical dimension in the Root technique. Part 2. Class II]	Excluded by title
168	Correction of deep overbite and gummy smile by using a mini-implant with a segmented wire in a growing Class II Division 2 patient	Excluded by title
169	Correction of severe class II skeletal discrepancy with fixed twin block and high pull headgear--a case report	Excluded by title
170	Correlation between chronological age and skeletal maturity in different malocclusions: A retrospective study	Excluded by title
171	Correlation between cone-beam CT images of the third and fourth cervical vertebrae and age in female skeletal class â… patients aged between 9 and 17 years in Qingdao]	Excluded by title
172	Correlations between dentoskeletal variables and deep bite in Class II Division 1 individuals	Excluded by title
173	CRANIOCERVICAL JUNCTION AS A FOCUS FOR CRANIOFACIAL GROWTH-STUDIES	Excluded by title
174	Craniocervical Posture in Children with Class I, II and III Skeletal Relationships	Excluded by title
175	Craniocervical Posture in Children with Class I, II and III Skeletal Relationships Class II, Division 1 Angle malocclusion with severe proclination of maxillary incisors	Excluded by title
176	Craniofacial abnormalities in a murine knock-out model of mucopolysaccharidosis I H: a computed tomography and anatomic study	Excluded by title
177	Craniofacial morphology in orthodontically treated patients of class III malocclusion with stable and unstable treatment outcomes	Excluded by title
178	Craniomaxillofacial Changes Using High-Pull J-Hook Headgear and Mini-Implant Anchorage in Adolescents: A Structural Superimposition Method	Excluded by title
179	Daily chewing gum exercise for stabilizing the vertical occlusion	Excluded by title
180	Dental and skeletal components of Class II open bite treatment with a modified Thurow appliance	Excluded by title
181	Dental and skeletal contributions to occlusal correction in patients treated with the high-pull headgear-activator combination	Excluded by title
182	Dental and skeletal effects after total arch distalization using modified C-palatal plate on hypo- and hyperdivergent Class II malocclusions in adolescents	Excluded by title
183	Dentoalveolar and skeletal changes associated with the pendulum appliance followed by fixed orthodontic treatment	Excluded by title
184	Dentofacial changes after orthodontic intervention with eruption guidance appliance in the early mixed dentition	Excluded by title
185	Dentoskeletal changes induced by the Jasper jumper and the activator-headgearcombination appliances followed by fixed orthodontic treatment	Excluded by title
186	Dentoskeletal comparison of miniscrew-anchored maxillary protraction with hybrid and conventional hyrax expanders: A randomized clinical trial	Excluded by title
187	Dentoskeletal Effects of Maxillary Protraction in Cleft Patients With Repetitive Weekly Protocol of Alternate Rapid Maxillary Expansions and Constrictions	Excluded by title
188	Dentoskeletal effects of Twin Block appliance in patients with Class II malocclusion	Excluded by title
189	Dentoskeletal features in mixed dentition children with displaced maxillary canines in a southern Italian population	Excluded by title
190	Dento-skeletal implications of Klippel-Feil syndrome a case report]	Excluded by title
191	Determinants of successful chincup therapy in skeletal class III malocclusion	Excluded by title
192	Determining the short-term effects of different maxillary protraction methods on pharyngeal airway dimensions	Excluded by title
193	Differences in craniofacial morphology between platybasic and nonplatybasic patients with velopharyngeal dysfunction and control subjects	Excluded by title
194	Differences in maxillary growth vector of skeletal class I with various vertical growth types before and after growth spurts]	Excluded by title
195	Differences of treatment outcomes between self-ligating brackets with microimplant and headgear anchorages in adults with bimaxillary protrusion	Excluded by title
196	Different approaches for management of Class III siblings: A long-term follow-up of two case reports	Excluded by title
197	Differential skeletal and dental effects after orthodontic treatment with bite jumping appliance or activator: a retrospective cephalometric study	Excluded by title
198	Dilemmas in Treatment of Recurrent Recalcitrant Dental Anterior Open Bite	Excluded by title
199	Dimensions of the craniocervical junction in longitudinal analysis of normal growth	Excluded by title
200	Distal movement of the first upper molars with pendulum appliance. Case report	Excluded by title
201	Does headgear treatment in young children affect the maxillary canine eruption path?	Excluded by title
202	Duration of the pubertal growth spurt in patients with increased craniofacial growth component in sagittal and vertical planes-retrospective and cross-sectional study	Excluded by title
203	Early class III treatment with hybrid rapid palatal expander combined with facemask	Excluded by title
204	Early Class III treatment with Hybrid-Hyrax - Facemask in comparison to Hybrid-Hyrax-Mentoplate - skeletal and dental outcomes	Excluded by title
205	Early headgear effect on the eruption pattern of maxillary second molars	Excluded by title
206	Early headgear effect on the eruption pattern of maxillary second molars	Excluded by title
207	Early orthodontic intervention followed by fixed appliance therapy in a patient with a severe Class III malocclusion and cleft lip and palate	Excluded by title
208	Early orthodontic treatment for Class II malocclusion reduces the chance of incisal trauma: Results of a Cochrane systematic review	Excluded by title
209	Early treatment of Class III incisor relationship using the chincap applian	Excluded by title
210	Early treatment of class III malocclusion with facemask	Excluded by title
211	Early treatment of class III malocclusion with Petit facemask therapy	Excluded by title

212	Early treatment of Class III: a long-term cohort study]	Excluded by title
213	Early treatment of pseudo-class III malocclusion with modified swallowing occlusal contact intercept appliance (S.O.C.I.A.)	Excluded by title
214	Early treatment of skeletal Class III open bite with the Tandem Appliance	Excluded by title
215	Early treatment of skeletal open-bite malocclusion	Excluded by title
216	Early treatment of vertical skeletal dysplasia: the hyperdivergent phenotype	Excluded by title
217	Early vs late orthodontic treatment of deepbite: a prospective clinical trial in growing subjects	Excluded by title
218	Edgewise therapy with cervical and intermaxillary traction--influence on the position of the bony chin	Excluded by title
219	Effect of Class III bone anchor treatment on airway	Excluded by title
220	Effect of mini-implants assisted Herbst in the treatment of permanent tooth early stage Angle Class II1 malocclusion of high angle mandibular retraction	Excluded by title
221	Effect of protraction facemask on the temporomandibular joint: a systematic review	Excluded by title
222	Effect of Treatment with Twin-Block Appliances on Body Posture in Class II Malocclusion Subjects: A Prospective Clinical Study	Excluded by title
223	Effectiveness of maxillary protraction using a hybrid hyrax-facemask combination: a controlled clinical study	Excluded by title
224	Effectiveness of maxillary protraction using facemask with or without maxillary expansion: a systematic review and meta-analysis	Excluded by title
225	Effects of a modified acrylic bonded rapid maxillary expansion appliance and vertical chin cap on dentofacial structures	Excluded by title
226	Effects of Class II activator and Class II activator high-pull headgear combination on the mandible: a 3-dimensional finite element stress analysis study	Excluded by title
227	Effects of Combined Rapid Maxillary Expansion and Facemask Therapy on the Mandibular Dental Arch in Mixed Dentition	Excluded by title
228	Effects of different vectors of forces applied by combined headgear	Excluded by title
229	Effects of facemask therapy on the mandibular retromolar space. A follow-up study	Excluded by title
230	Effects of facemask treatment anchored with miniplates after alternate rapid maxillary expansions and constrictions; a pilot study	Excluded by title
231	Effects of facemasks versus intraoral appliances in treating maxillary deficiency in growing patients: A systematic review and meta-analysis	Excluded by title
232	Effects of headgear Herbst and mandibular step-by-step advancement versus conventional Herbst appliance and maximal jumping of the mandible	Excluded by title
233	Effects of hybrid-Hyrax, Alt-RAMEC and miniscrew reinforced heavy Class III elastics in growing maxillary retrusive patients. A four-year follow-up pilot study	Excluded by title
234	Effects of mandibular protraction appliance associated to fixed appliance in adults	Excluded by title
235	Effects of pendulum appliance versus clear aligners in the vertical dimension during Class II malocclusion treatment: a randomized prospective clinical trial	Excluded by title
236	Effects of retraction of anterior teeth and initial soft tissue variables on lip changes in Japanese adults	Excluded by title
237	Effects of Sabbagh Universal Spring 2 fixed functional appliance on class II/1 patients at their postpubertal-peak growth period compared with the extraction method	Excluded by title
238	Effects of the headgear-activator Teuscher appliance in the treatment of Class II Division 1 malocclusion: a geometric morphometric study	Excluded by title
239	Effects of the Herbst appliance in growing orthodontic patients with different underlying vertical patterns	Excluded by title
240	Effects of the reciprocal mini-chin cup appliance	Excluded by title
241	Effects of treatment with a combined maxillary protraction and chincap appliance in skeletal Class III patients with different vertical skeletal morphologies	Excluded by title
242	Effects of vertical chincap therapy on the mandibular morphology in open-bite patients	Excluded by title
243	Efficacy of clear aligners in producing molar distalization: Systematic review	Excluded by title
244	Eight-year stability of a severe skeletal anterior open bite with a hyperdivergent growth pattern treated with an edgewise appliance and chin cup therapy	Excluded by title
245	Electromyographic evaluation of masticatory, neck, and trunk muscle activity in patients with posterior crossbites	Excluded by title
246	Endoscopic endonasal surgical management of chondrosarcomas with cerebellopontine angle extension	Excluded by title
247	En-masse retraction dependent on a temporary skeletal anchorage device without posterior bonding or banding in an adult with severe bidentoalveolar protrusion: Seven years posttreatment	Excluded by title
248	Esthetic facial surgery]	Excluded by title
249	Ethnic differences in craniofacial and upper spine morphology in children with skeletal Class II malocclusion	Excluded by title
250	Etiology and treatment of cervical kyphosis: state of the art review-a narrative review	Excluded by title
251	Evaluation of cervical posture of children in skeletal class I, II, and III	Excluded by title
252	Evaluation of cervical spine posture after functional therapy with FR-2: A longitudinal study	Excluded by title
253	Evaluation of cervical spine posture after functional therapy with twin-block appliances: A retrospective cohort study	Excluded by title
254	Evaluation of changes in the vertical facial dimension with different anchorage systems in extraction and non-extraction subjects treated by Begg fixed appliances: a retrospective study	Excluded by title
255	Evaluation of masseter muscle in different vertical skeletal patterns in growing patients	Excluded by title
256	Evaluation of maxillary protraction and fixed appliance therapy in Class III patients	Excluded by title
257	Evaluation of posttreatment changes in Class II Division 1 patients after nonextraction orthodontic treatment: Cephalometric and model analysis	Excluded by title
258	Evaluation of Protraction Face-Mask Therapy on the Craniofacial and Upper Airway Morphology in Unilateral Cleft Lip and Palate	Excluded by title
259	Evaluation of sagittal airway dimensions after face mask therapy with rapid maxillary expansion in Class III growing patients	Excluded by title
260	Evaluation of skeletal maturity in the cervical vertebrae and hand-wrist in relation to vertical facial types	Excluded by title
261	Extraction of the lateral incisors to treat maxillary protrusion: quantitative evaluation of the stomatognathic functions	Excluded by title
262	Extra-oral retraction mechanics: a review	Excluded by title
263	Extraoral vs intraoral appliance for distal movement of maxillary first molars: a randomized controlled trial	Excluded by title
264	Face mask therapy with skeletal anchorage: A possible alternative to orthognathic surgery	Excluded by title
265	Facemask performance during maxillary protraction: a finite element analysis (FEA) evaluation of load and stress distribution on Delaire facemask	Excluded by title
266	Factors associated with long-term vertical skeletal changes induced by facemask therapy in patients with Class III malocclusion	Excluded by title
267	Features and treatment of skeletal class III malocclusion with severe lateral mandibular shift and asymmetric vertical dimension	Excluded by title
268	Forsus Nitinol Flat Spring and Jasper Jumper corrections of Class II division 1 malocclusions	Excluded by title
269	Functional and fixed orthodontic treatment in a child with cerebral palsy	Excluded by title
270	General Practice and the Community: Research on health service, quality improvements and training. Selected abstracts from the EGPRN Meeting in Vigo, Spain, 17-20 October 2019 Abstracts	Excluded by title
271	Gingival Crevicular Fluid Cytokine Levels in Response to Orthodontic Forces	Excluded by title
272	Growth and alignment of the pediatric subaxial cervical spine following rigid instrumentation and fusion: a multicenter study of the Pediatric Craniocervical Society	Excluded by title
273	GROWTH OF ACELLULAR EXTRINSIC FIBER CEMENTUM (AEFC) AND DENSITY OF INSERTING FIBERS IN HUMAN PREMOLARS OF ADOLESCENTS	Excluded by title
274	Gunel E Comparison of two maxillary protraction protocols: tooth-borne versus bone-anchored protraction facema	Excluded by title
275	Gut Microbiota From SjÃ¶gren syndrome Patients Causes Decreased T Regulatory Cells in the Lymphoid Organs and Desiccation-Induced Corneal Barrier Disruption in Mice	Excluded by title
276	Head posture in cleft lip and palate patients with oronasal fistula and its relationship with craniofacial morphology	Excluded by title
277	Head posture in lateral roentgen image]	Excluded by title
278	Head posture in obstructive sleep apnoea	Excluded by title
279	Headgear-free molar distalization]	Excluded by title
280	HEALTH-RELATED QUALITY OF LIFE IN A PATIENT FOLLOWING ANEURYSMOTOMY OF RARE MEGA-GIANT ANEURYSM OF THE COMMON CAROTID ARTERY	Excluded by title
281	Herbst treatment in late adolescence: clinical, electromyographic, kinesiographic, and radiographic analysis of one case	Excluded by title
282	Heritability of mandibular cephalometric variables in twins with completed craniofacial growth	Excluded by title
283	High pull headgear with J-hooks to upper removable appliances	Excluded by title
284	Hormone replacement therapy after treatment for a gynaecological malignancy	Excluded by title
285	Hyrax application as a tooth-borne distractor for maxillary advancement	Excluded by title
286	Illustration of vertical control in hyperdivergence with the Tweed technique]	Excluded by title
287	Image measurements of os odontoideum in children	Excluded by title
288	Immediate skeletal and dentoalveolar effects of the crown- or banded type Herbst appliance on Class II division 1 malocclusion	Excluded by title
289	Impact of Cervical Sagittal Alignment Parameters on Neck Disability	Excluded by title
290	Impact of molar teeth distalization with clear aligners on occlusal vertical dimension: a retrospective study	Excluded by title
291	Improvement of masticatory function after orthodontic treatment. Two case reports	Excluded by title
292	Incisor torque by means of a modified Teuscher activator. Three case reports	Excluded by title
293	Incremental effects of facemask therapy associated with intermaxillary mechanics	Excluded by title
294	Individual response to treatments using Teuscher activator]	Excluded by title
295	INFLUENCE OF OCCLUSAL VERTICAL DIMENSION ON CERVICAL SPINE MOBILITY IN SPORTS SUBJECTS	Excluded by title
296	Influence of orthopedic treatment on hard and soft facial structures of individuals presenting with Class II, Division 1 malocclusion: a comparative study	Excluded by title
297	Influence of skeletal class in the morphology of cervical vertebrae: A study using cone beam computed tomography	Excluded by title
298	Infrazygomatic Crest Miniscrews and Zygomatic Miniplates: a Randomized Clinical Trial	Excluded by title
299	Initial and late treatment effects of headgear-Herbst appliance with mandibular step-by-step advancement	Excluded by title
300	Interactions of hard tissues, soft tissues, and growth over time, and their impact on orthodontic diagnosis and treatment planning	Excluded by title
301	Interceptive orthopedics for the correction of maxillary transverse and sagittal deficiency in the early mixed dentition period	Excluded by title
302	Internal fixation for osteomyelitis of cervical spine: the issue of persistence of culture positive infection around the implants	Excluded by title
303	Intraoperative Use of Cone-Beam Computed Tomography in the Treatment of Atlantoaxial Rotatory Subluxation	Excluded by title
304	Intrusion of posterior teeth using miniplates: intrusive mechanics is not the same as intrusion force	Excluded by title
305	Investigating the effect of micro-osteoperforation on the rate of tooth movements	Excluded by title
306	Invisalign(Â®) treatment of patients with craniomandibular disorders	Excluded by title
307	Locating the center of resistance of maxillary anterior teeth retracted by Double J Retractor with palatal miniscrews	Excluded by title
308	Longitudinal growth changes in untreated subjects with Class II Division 1 malocclusion	Excluded by title
309	Longitudinal soft-tissue profile changes in adolescent Class I subjects	Excluded by title
310	Long-term anteroposterior and vertical maxillary changes in skeletal class II patients treated with slow and rapid maxillary expansion	Excluded by title
311	Long-term application of chincup force alters the morphology of the dolichofacial Class III mandible	Excluded by title
312	Long-term changes in dentoskeletal pattern in a case with Beckwith-Wiedemann syndrome following tongue reduction and orthodontic treatment	Excluded by title
313	Long-term changes in pharyngeal airway dimensions following activator-headgearand fixed appliance treatment	Excluded by title
314	Long-term comparison of treatment outcome and stability of Class II patients treated with functional appliances versus bilateral sagittal split ramus osteotomy	Excluded by title
315	Long-Term Dentoskeletal Changes with the Bionator, Herbst, Twin Block, and MARA Functional Appliances	Excluded by title
316	Long-term effects of chin-cap therapy on the temporomandibular joints	Excluded by title
317	Long-term efficacy of reverse pull headgear therapy	Excluded by title
318	Long-term evaluation of the molar movements following Pendulum and fixed appliances	Excluded by title

319	Long-term follow-up of a patient with achondroplasia treated with an orthodontic approach	Excluded by title
320	Long-term maintenance of cervical alignment after occipitocervical and atlantoaxial screw fixation in young children	Excluded by title
321	Long-term mandibular skeletal and dental effects of standard edgewise treatment	Excluded by title
322	Long-term maxillary three dimensional changes following maxillary protraction with or without expansion: A systematic review and meta-analysis	Excluded by title
323	Long-term orthodontic and surgical treatment and stability of a patient with Beckwith-Wiedemann syndrome	Excluded by title
324	Long-term outcome in a patient with a dentoskeletal open-bite malocclusion treated without extraction	Excluded by title
325	Long-term profile changes associated with successfully treated extraction and nonextraction Class II Division 1 malocclusions	Excluded by title
326	Long-term results of skeletal profile changes occurring during chincap therapy in male Japanese skeletal Class III cases]	Excluded by title
327	Long-term skeletal and dental effects and treatment timing for functional appliances in Class II malocclusion	Excluded by title
328	Long-term stability of maxillary protraction therapy in Class III patients with complete unilateral cleft lip and palate	Excluded by title
329	Long-term survival of adult cancer patients from a psychosomatic perspective - literature review and consequences for future research	Excluded by title
330	Lower first-molar extractions and directional forces in high-angle class III treatment	Excluded by title
331	Lower incisor changes on basal bone and in relation to the lower face: combined growth and treatment effects in the late mixed-dentition	Excluded by title
332	Management of Anterior Open Bite and Skeletal Class II Hyperdivergent Patient with Clear Aligner Therapy	Excluded by title
333	Management of severe Class II division 1 malocclusion: a case report	Excluded by title
334	Management of Severe Class II Malocclusion With Sequential Fixed Functional and Orthodontic Appliances: A Case for MOrthRCSEd Examination	Excluded by title
335	Mandibular behavior with slow and rapid maxillary expansion in skeletal Class II patients - A long-term study	Excluded by title
336	Mandibular changes in skeletal class II patients treated with Kloehn cervical headgear	Excluded by title
337	Mandibular growth direction with conventional Class II nonextraction treatment	Excluded by title
338	Mandibular molar displacement secondary to the use of forces to retract the maxilla	Excluded by title
339	Maxillary displacement with mandibular maxi-propulsion in the treatment of Class II division 1]	Excluded by title
340	Maxillary protraction in patients with cleft lip and palate in mixed dentition: cephalometric evaluation after completion of growth	Excluded by title
341	Maxillary protraction using a hybrid hyrax-facemask combination	Excluded by title
342	Maxillary protraction using orthodontic miniplates in correction of Class III malocclusion during growth	Excluded by title
343	Maxillofacial intraoral distraction osteogenesis followed by elastic traction in cleft maxillary deformity	Excluded by title
344	Meta-analysis of the efficacy of bone anchorage and maxillary facemask protraction devices in treating skeletal class â…¢ malocclusion in adolescents]	Excluded by title
345	Methodological quality and outcome of systematic reviews reporting on orthopaedic treatment for class III malocclusion: Overview of systematic reviews	Excluded by title
346	Mid-line clefts of the cervical vertebrae - an incidental finding arising from cone beam computed tomography of the dental patient	Excluded by title
347	Mid-term follow up effectiveness of facemask treatment in class III malocclusion: A systematic review	Excluded by title
348	Mixed dentition case report	Excluded by title
349	Mixed dentition treatment with cervical traction and lower lingual arch	Excluded by title
350	Modification protocol for an early class III treatment using 3 miniplates for bone-anchored maxillary orthopedic traction	Excluded by title
351	Modified fixed nanobite tandem appliance for rapid correction of developing Class III malocclusion	Excluded by title
352	Modified maxillary protraction headgear for the correction of class III skeletal malocclusion with anterior open bite	Excluded by title
353	Modified SEC III protocol: vertical control related to patients' compliance with the chincup	Excluded by title
354	Modified Thurow appliance: a clinical alternative for correcting skeletal open bite	Excluded by title
355	Monitoring growth during orthodontic treatment	Excluded by title
356	Morphologic changes in the transverse dimension using the FrÃ¤nkel appliance	Excluded by title
357	Morphological Characteristics of the Cranial Base of Early Angle's Class II Division 1 Malocclusion in Permanent Teeth	Excluded by title
358	Mouth Rehabilitation of a Patient with Severe Deep Bite: A Clinical Report	Excluded by title
359	Natural changes of the maxillary first molars in adolescents with skeletal Class II malocclusion	Excluded by title
360	New perspective on Herbst therapy for skeletal Class II malocclusions: a proposal for maxillary protrusion management	Excluded by title
361	Noncarious cervical lesions: Morphology and progression, prevalence, etiology, pathophysiology, and clinical guidelines for restoration	Excluded by title
362	Nonextraction and nonsurgical treatment of an adult with skeletal Class II open bite with severe retrognathic mandible and temporomandibular disorders	Excluded by title
363	Nonextraction treatment of a high-angle Class II case with a modified Herbst appliance	Excluded by title
364	Nonextraction treatment of a skeletal Class III adolescent girl with expansion and facemask: long-term stability	Excluded by title
365	Nonextraction treatment of a skeletal Class III malocclusion	Excluded by title
366	Nonsurgical Correction of Severe Anterior Openbite Malocclusion	Excluded by title
367	Nonsurgical correction using miniscrew-assisted vertical control of a severe high angle with mandibular retrusion and gummy smile in an adult	Excluded by title
368	Nonsurgical maxillary orthopedic protraction treatment for an adult patient with hyperdivergent facial morphology, Class III malocclusion, and bilateral crossbite	Excluded by title
369	Noonan syndrome: a case report	Excluded by title
370	Obstructive sleep apnea subtypes by cluster analysis	Excluded by title
371	Occlusal Plane Changes After Molar Distalization With a Pendulum Appliance in Growing Patients with Class II Malocclusion: A Retrospective Cephalometric Study	Excluded by title
372	Occlusal stability after Herbst treatment of patients with retrognathic and prognathic facial types A pilot study	Excluded by title
373	Ocular hypertelorism in an orthodontic patient	Excluded by title
374	Open bite and and therapeutic strategy]	Excluded by title
375	Open-bite treatment with vertical control and tongue reeducation	Excluded by title
376	Oral breathing and head posture	Excluded by title
377	Orthodontic and orthopaedic approach in the treatment of skeletal open bite	Excluded by title
378	Orthodontic and orthopedic effects of Activator, Activator-HG combination, and Bass appliances: a comparative study	Excluded by title
379	Orthodontic compensation in skeletal Class III malocclusion: a case report	Excluded by title
380	Orthodontic Consideration in Patients with Beta-Thalassemia Major: Case Report and Literature Review	Excluded by title
381	Orthodontic cooperation	Excluded by title
382	Orthodontic management of the short face patient	Excluded by title
383	Orthodontic traction of impacted canines: Concepts and clinical application	Excluded by title
384	Orthodontic treatment for jaw deformities in cleft lip and palate patients with the combined use of an external-expansion arch and a facial mask	Excluded by title
385	Orthodontic treatment for prominent upper front teeth (Class II malocclusion) in children and adolescents	Excluded by title
386	Orthodontic treatment in the severely compromised periodontal patient	Excluded by title
387	Orthodontic treatment with growth hormone therapy in a girl of short stature	Excluded by title
388	Orthopaedic approach of maxillary hypoplasia treatment	Excluded by title
389	Orthopaedic treatment efficiency in skeletal Class III malocclusions in young patients: RME-face mask versus TSME	Excluded by title
390	Orthopedic correction of Class III malocclusion: retention and phase II therapy	Excluded by title
391	Orthopedic Correction of Growing Hyperdivergent, Retrognathic Patients With Miniscrew Implants	Excluded by title
392	Osseointegrated implants with pendulum springs for maxillary molar distalization: A cephalometric study	Excluded by title
393	Outcomes of early versus late treatment of severe Class II high-angle patients	Excluded by title
394	Palatal configuration in Class II Division 1 malocclusion: A longitudinal study	Excluded by title
395	Performance of UK National Health Service compared with other high income countries: observational study	Excluded by title
396	Pharyngeal airway and hyoid bone position changes of skeletal anchored Forsus Fatigue Resistant Device and activator appliances	Excluded by title
397	Pharyngeal airway dimensions after chin cup treatment in Class III malocclusion subjects	Excluded by title
398	Physical properties of root cementum: part 24. Root resorption of the first premolars after 4 weeks of occlusal trauma	Excluded by title
399	Ponticulus posticus in a cohort of orthodontic children and adolescent patients with different sagittal skeletal anomalies: a comparative cone beam computed tomography investigation	Excluded by title
400	Postburn Neck Anterior Contracture Treatment in Children With Scar-Fascial Local Trapezoid Flaps: A New Approach	Excluded by title
401	Postpubertal assessment of treatment timing for maxillary expansion and protraction therapy followed by fixed appliances	Excluded by title
402	Posttreatment changes of skeletal morphology following treatment aimed at restriction of maxillary growth	Excluded by title
403	Predicting changes in mandibular length and total anterior	Excluded by title
404	Predicting the Occurrence of Postoperative Distal Junctional Kyphosis in Cervical Deformity Patients	Excluded by title
405	Predicting vertical growth of the mandibular ramus via hand-wrist radiographs	Excluded by title
406	Prevalence of ponticulus posticus among patients with different dental malocclusions by digital lateral cephalogram: a comparative study	Excluded by title
407	Primary squamous cell carcinoma of parotid gland with sialo-cutaneous fistula: a rare clinical case	Excluded by title
408	Protraction of the maxillofacial complex	Excluded by title
409	Protraction--it's use and abuse]	Excluded by title
410	Quantitative analysis of the orthodontic and orthopedic effects of maxillary traction	Excluded by title
411	Quasi-Least Squares Regression Method with Dentistry Data	Excluded by title
412	Radiographic assessment of skeletal maturation stages for orthodontic patients: Hand-wrist bones or cervical vertebrae?	Excluded by title
413	Radiographic evaluation of dental and cervical vertebral development for age estimation in a young Brazilian population	Excluded by title
414	Realities of craniofacial growth modification	Excluded by title
415	Redirecting mandibular growth through orthodontic dentoalveolar height development in growing patients with Class III malocclusion undergoing maxillary orthopedic protraction	Excluded by title
416	Relationship between head posture and dentofacial morphology in patients with TMJ osteoarthritis/osteoarthrosis	Excluded by title
417	Relationship between malocclusion severity and treatment success rate in Class II nonextraction therapy	Excluded by title
418	Reliability of Growth Indicators and Efficiency of Functional Treatment for Skeletal Class II Malocclusion: Current Evidence and Controversies	Excluded by title
419	Repositioning of premaxilla in bilateral cleft lip and palate using a "J-hook headgear"	Excluded by title
420	Retreatment of a patient with Marfan syndrome and severe root resorption	Excluded by title
421	Retrospective 25-year follow-up of treatment outcomes in angle Class III patients : Early versus late treatment	Excluded by title
422	Rotational effects of Class II Division 1 treatment with the Herbst appliance and fixed appliances in growing subjects with different vertical patterns	Excluded by title
423	Sagittal airway dimensions following maxillary protraction: a pilot study	Excluded by title
424	Sagittal and vertical changes after treatment of Class II Division 1 malocclusion according to the Cetlin method	Excluded by title
425	Sagittal and Vertical Craniofacial Growth Pattern and Timing of Circumpubertal Skeletal Maturation: A Multiple Regression Study	Excluded by title
426	Sagittal and vertical occlusal cephalometric analyses of Pancherz: Norms for Chinese children	Excluded by title
427	Sagittal spinal posture in relation to craniofacial morphology	Excluded by title

428	Schwannoma of the sympathetic trunk: A case report	Excluded by title
429	Serial extraction in class II malocclusions	Excluded by title
430	Severe anterior open-bite malocclusion	Excluded by title
431	Severe Class II anterior deep bite malocclusion treated with a C-lingual retractor	Excluded by title
432	Severe high Angle Class II Division 1 malocclusion with vertical maxillary excess and gummy smile: a case report	Excluded by title
433	Sexual dimorphism in the long-term stability (10 years) of skeletal Class III treatment	Excluded by title
434	Short-term effects of a modified Alt-RAMEC protocol for early treatment of Class III malocclusion: a controlled study	Excluded by title
435	Short-term sagittal changes of the upper and lower jaws in patients treated with acrylic-splint rapid palatal expander before growth peak	Excluded by title
436	Skeletal and dental effects of a mini maxillary protraction appliance	Excluded by title
437	Skeletal and dental effects of Class III orthopaedic treatment: a systematic review and meta-analysis	Excluded by title
438	Skeletal and dentoalveolar effects of hybrid rapid palatal expansion and facemask treatment in growing skeletal Class III patients	Excluded by title
439	Skeletal cephalometric modifications in growing patients submitted to functional appliances treatment	Excluded by title
440	Skeletal changes associated with extraoral appliance therapy: an evaluation of 200 consecutively treated cases	Excluded by title
441	Skeletal changes of maxillary protraction in patients exhibiting skeletal class III malocclusion: a comparison of three skeletal maturation groups	Excluded by title
442	Skeletal maturation and the location of the mandibular foramen within the ramus mandibulae	Excluded by title
443	Skeletal maturation in different anteroposterior and vertical skeletal growth patterns in female subjects	Excluded by title
444	Skeletal response to maxillary protraction in patients with cleft lip and palate before age 10 years	Excluded by title
445	Skeletal, dental and soft tissue changes in Class III patients treated with fixed appliances and lower premolar extractions	Excluded by title
446	Soft and hard tissue changes after maxillary protraction with skeletal anchorage implant in treatment of Class III malocclusion]	Excluded by title
447	Soft and hard tissue profile changes after rapid maxillary expansion and face mask therapy	Excluded by title
448	SOFT TISSUE CHANGES IN PATIENTS WITH DENTOALVEOLAR PROTRUSION TREATED WITH MAXIMUM ANCHORAGE: A SYSTEMATIC REVIEW AND META-ANALYSIS	Excluded by title
449	Soft tissue facial profile in Class III malocclusion: long-term post-pubertal effects produced by the Face Mask Protocol	Excluded by title
450	Soft Tissue Profile Changes Following Orthodontic Treatment in Patients With Unilateral Cleft Lip and Palate	Excluded by title
451	Some characteristics of orthodontic treatment in high mandibular cases]	Excluded by title
452	Stability and relapse of maxillary anterior crowding treatment in class I and class II Division 1 malocclusions	Excluded by title
453	Stability of Class II malocclusion treatment with the distal jet followed by fixed appliances	Excluded by title
454	Stability of orthodontic treatment outcome: Follow-up until 10 years postretention	Excluded by title
455	Stability of the mandible after surgical correction of skeletal class III malocclusion in 50 patients	Excluded by title
456	Stability prediction of early orthopedic treatment in Class III malocclusion: morphologic discriminant analysis	Excluded by title
457	Stepwise advancement versus maximum jumping with headgear activator	Excluded by title
458	Stress distribution in maxillary first molar periodontium using straight pull headgear with vertical and horizontal tubes: A finite element analysis	Excluded by title
459	Success rate, costs and long-term stability of treatment with activator/headgear combinations	Excluded by title
460	Superimpositional assessment of treatment-associated changes in the temporomandibular joint and the mandibular symphysis	Excluded by title
461	Surgical Accuracy of Positioning the Maxilla in Patients With Skeletal Class II Malocclusion Using Computer-Aided Design and Computer-Aided Manufacturing-Assisted Orthognathic Surgery	Excluded by title
462	Surgical management of extracranial internal carotid artery aneurysms	Excluded by title
463	Surgically-assisted orthopedic protraction of the maxilla in cleft lip and palate patients	Excluded by title
464	Surgical-orthodontic correction of open-bite deformity	Excluded by title
465	Surgical-orthodontic treatment of Class III malocclusion with agenesis of lateral incisor and unerupted canine	Excluded by title
466	Symphysis morphology and mandibular alveolar bone thickness in patients with Î²-thalassemia major and different growth patterns	Excluded by title
467	Temporary occipital fixation in young children with severe cervical-thoracic spinal deformity	Excluded by title
468	Temporomandibular disorders in relation to craniofacial dimensions, head posture and bite force in children selected for orthodontic treatment	Excluded by title
469	Temporomandibular Joint Disk Displacements in Class II Malocclusion and Cervical Spine Alterations: Systematic Review and Report of a Hypodivergent Case with MRI Bone and Soft Tissue Changes	Excluded by title
470	The accuracy of maxillary positioning using digital model planning and 3D printed wafers in bimaxillary orthognathic surgery	Excluded by title
471	The analysis of the changes of tongue shape and position, hyoid position in Class II, division 1 malocclusion treated with functional appliances	Excluded by title
472	The best time for orthodontic treatment for Polish children based on skeletal age analysis in accordance to refund policy of the Polish National Health Fund (NFZ)	Excluded by title
473	The BOS MOrth Cases Prize 2009	Excluded by title
474	The cervical vertebrae maturation (CVM) method cannot predict craniofacial growth in girls with Class II malocclusion	Excluded by title
475	The class II malocclusion: differential diagnosis and clinical application of activators, extraoral traction, and fixed appliances	Excluded by title
476	The clinical effect evaluation of a new implant nail assisted remote movement of invisible molars method with palatal mini-screw	Excluded by title
477	The clinical management of open-bite malocclusions	Excluded by title
478	The cross-sectional effects of ribbon arch wires on Class II malocclusion intermaxillary traction: a three-dimensional finite element anal	Excluded by title
479	The dental and skeletal effects of the jumping-the-bite plate and high-pull headgear combination. A clinical study of treated patients]	Excluded by title
480	The Don Spring Memorial Oration--Part II: early management of the developing Class III malocclusion	Excluded by title
481	The effect of a modified reverse headgear force applied with a facebow on the dentofacial structures	Excluded by title
482	The effect of an anterior biteplate on dental and skeletal Class II correction using headgears: a cephalometric study	Excluded by title
483	The effect of cervical headgear on patients with high or low mandibular plane angles and the myth of posterior mandibular rotation	Excluded by title
484	The effect of dental occlusal disturbances on the curvature of the vertebral spine in rats	Excluded by title
485	The effect of headgear on upper third molars: a retrospective longitudinal study	Excluded by title
486	The effect of stepwise increases in vertical dimension of occlusion on isometric strength of cervical flexors and deltoid muscles in nonsymptomatic females	Excluded by title
487	The Effect of Timing on Orthodontic Treatment	Excluded by title
488	The effect of treatment with the Bass appliance on skeletal Class II malocclusions	Excluded by title
489	The effect of treatment with the Bass appliance on skeletal Class II malocclusions: a cephalometric investigation	Excluded by title
490	The effect of zigzag elastics in the treatment of Class II division 1 malocclusion subjects with hypo- and hyperdivergent growth patterns. A pilot study	Excluded by title
491	The effectiveness of alternating rapid maxillary expansion and constriction combined with maxillary protraction in the treatment of patients with a class III malocclusion: a systematic review and meta-analysis	Excluded by title
492	The effects of activator treatment on the craniofacial structures of Class II division 1 patients	Excluded by title
493	The effects of maxillary protraction and its long-term stability--a clinical trial in Chinese adolescents	Excluded by title
494	The Effects of Maxillary Protraction with or without Rapid Maxillary Expansion and Age Factors in Treating Class III Malocclusion: A Meta-Analysis	Excluded by title
495	The effects of sex, skeletal age, and sagittal skeletal pattern on pharyngeal airway dimensions and related structures in growing Thai orthodontic patients	Excluded by title
496	The effects of the headgear therapy on the airway dimensions in patients with class II malocclusion: A systematic review	Excluded by title
497	The effects, limitations, and long-term dentofacial adaptations to treatment with the Herbst appliance	Excluded by title
498	The efficacy of maxillary protraction protocols with the micro-implant-assisted rapid palatal expander (MARPE) and the novel N2 mini-implant-a finite element study	Excluded by title
499	The Endonasal Endoscopic Approach to Pathologies of the Anterior Craniocervical Junction: Analytical Review of Cases Treated at Four European Neurosurgical Centres	Excluded by title
500	THE EVALUATION OF REPRODUCIBILITY OF OPERATION PLAN WITH THE COMPARISON BETWEEN STO AND POST-OPERATIVE JAW POSITION IN ORTHOGNATHIC SURGERY	Excluded by title
501	The Grummons face mask as an early treatment modality within a class III therapy concept	Excluded by title
502	The hyoid bone position in adult individuals with open bite and normal occlusion	Excluded by title
503	The Lynn Maxilla Rotator Combination Appliance and Lynn Archial Face Bow	Excluded by title
504	The maxillary orthopedic splint	Excluded by title
505	The OPA: a device for vertical control	Excluded by title
506	The relationship between sagittal upper airway size and surrounding skeletal structure with Delaire cephalometric analysis]	Excluded by title
507	The role of a high pull headgear in counteracting side effects from intrusion of the maxillary anterior segment	Excluded by title
508	The treatment of severe 'gummy' Class II division 1 malocclusion using the maxillary intrusion splint	Excluded by title
509	The William Houston Gold Medal of the Royal College of Surgeons of Edinburgh 2009	Excluded by title
510	The'SOS activator:'A Novel Functional Device Combined with Fixed Appliances for the Correction of Class II Malocclusion	Excluded by title
511	Thin-plate spline graphical analysis of the mandible in mandibular prognathism	Excluded by title
512	Three-dimensional alterations in pharyngeal airway and maxillary sinus volumes in Class III maxillary deficiency subjects undergoing orthopedic facemask treatment	Excluded by title
513	Three-dimensional analysis of maxillary changes associated with facemask and rapid maxillary expansion compared with bone anchored maxillary protraction	Excluded by title
514	Three-dimensional assessment of mandibular and glenoid fossa changes after bone-anchored Class III intermaxillary traction	Excluded by title
515	Three-Dimensional Assessment of Pharyngeal Airway Space by MRI in Class II Division 1 Patients Treated by Twin Block Appliance	Excluded by title
516	Three-dimensional computed tomography in obstructive sleep apneics treated by maxillomandibular advancement	Excluded by title
517	Three-dimensional dental arch and palatal form changes after extraction and nonextraction treatment. Part 2. Palatal volume and height	Excluded by title
518	Three-dimensional dental model analysis of treatment outcomes for protrusive maxillary dentition: comparison of headgear, miniscrew, and miniplate skeletal anchorage	Excluded by title
519	Three-dimensional diagnosis and management of Class II malocclusion in the mixed dentition	Excluded by title
520	Three-dimensional effects of the mini-implant-anchored Forsus Fatigue Resistant Device: A randomized controlled trial	Excluded by title
521	Three-dimensional effects of the mini-implant-anchored Forsus Fatigue Resistant Device: a randomized controlled trial	Excluded by title
522	Three-dimensional evaluation of skeletal and dental effects of treatment with maxillary skeletal expansion	Excluded by title
523	Three-dimensional evaluation of tooth movement in Class II malocclusions treated without extraction by orthodontic mini-implant anchorage	Excluded by title
524	Three-dimensional skeletal and dentoalveolar sagittal and vertical changes associated with cantilever Herbst appliance in prepubertal patients with Class II malocclusion	Excluded by title
525	Timing for effective application of anteriorly directed orthopedic force to the maxilla	Excluded by title
526	Timing of myofunctional appliance therapy	Excluded by title
527	Tomographic evaluation of buccal bone in different skeletal patterns and incisors inclination	Excluded by title
528	Traditional face-bow transfer versus three-dimensional virtual reconstruction in orthognathic surgery	Excluded by title
529	Treatment and posttreatment craniofacial changes after rapid maxillary expansion and facemask therapy	Excluded by title
530	Treatment and posttreatment effects of acrylic splint Herbst appliance therapy	Excluded by title
531	Treatment and post-treatment effects of facemask therapy on the sagittal pharyngeal dimensions in Class III subjects	Excluded by title
532	Treatment changes of hypo- and hyperdivergent Class II Herbst patients	Excluded by title
533	Treatment effect of bone-anchored maxillary protraction in growing patients compared to controls: a systematic review with meta-analysis	Excluded by title

534	Treatment effects and anchorage potential of sliding mechanics with titanium screws compared with the Tweed-Merrifield technique	Excluded by title
535	Treatment effects of bonded RME and verticalpull chincup followed by fixed appliance in patients with increased vertical dimension	Excluded by title
536	Treatment effects of FrÃ¤nkel, activator and extraoral traction appliances	Excluded by title
537	Treatment Effects of Removable Functional Appliances in Pre-Pubertal and Pubertal Class II Patients: A Systematic Review and Meta-Analysis of Controlled Studies	Excluded by title
538	Treatment effects of Reverse Twin-Block and Reverse Pull Face Mask on craniofacial morphology in early and late mixed dentition children	Excluded by title
539	Treatment effects of the bionator and high-pull facebow combination followed by fixed appliances in patients with increased vertical dimensions	Excluded by title
540	Treatment effects of the Herbst appliance	Excluded by title
541	Treatment effects of the light-force chincup	Excluded by title
542	Treatment effects produced by the Twin-block appliance and the FR-2 appliance of Frankel compared with an untreated Class II sample	Excluded by title
543	Treatment in the deciduous dentition: four clinical cases	Excluded by title
544	Treatment of a Case of Skeletal Class II Malocclusion with Temporomandibular Joint Disorder Using Miniscrew Anchorage	Excluded by title
545	Treatment of a Class II division 1 anterior open bite malocclusion	Excluded by title
546	Treatment of Class II high angle malocclusions with the Herbst appliance: a cephalometric investigation	Excluded by title
547	Treatment of Deep Bite Malocclusions	Excluded by title
548	Treatment Possibilities of Diff erent Skeletal Anchorage Systems in View of Failures and Risk Factors	Excluded by title
549	Treatment response and long-term dentofacial adaptations to maxillary expansion and protraction	Excluded by title
550	Treatment response to maxillary expansion and protraction	Excluded by title
551	Treatment stability after total maxillary arch distalization with modified C-palatal plates in adults	Excluded by title
552	Treatment times of Class II malocclusion: four premolar and non-extraction protocols	Excluded by title
553	Treatment timing for Twin-block therapy	Excluded by title
554	True molar intrusion attained during orthodontic treatment: A systematic review	Excluded by title
555	Two Class II, division 1 patients with congenitally missing lower central incisors	Excluded by title
556	Two different applications of Class II elastics with nonextraction segmental techniques	Excluded by title
557	Two-phase orthodontic treatment of a complex malocclusion: giving up efficiency in favor of effectiveness, quality of life, and functional rehabilitation?	Excluded by title
558	Two-phase treatment of skeletal class II malocclusion with the combination of the twin-block appliance and high-pull headgear	Excluded by title
559	Upper Airway Characteristics in Young Individuals With Class II Division 1 Malocclusion: A Retrospective Inter-Ethnic Cephalometric Comparison	Excluded by title
560	Upper spine morphology in hypophosphatemic rickets and healthy controls: a radiographic study	Excluded by title
561	Uprighting the mandibular molars stimulates mandibular growth during treatment of class II malocclusion	Excluded by title
562	Use of functional bite plate (the J.A. Cervera PFB) in the treatment of skeletal and dental open bite]	Excluded by title
563	Variations of total vertical maxillary excess	Excluded by title
564	Verrucous carcinoma of the larynx. - A study of its pathologic anatomy	Excluded by title
565	Vertical control as an important ingredient in the treatment of severe sagittal discrepancies	Excluded by title
566	Vertical development of the face and cervical spine. Diagnostic and therapeutic significance in orthodontics and maxillofacial surgery]	Excluded by title
567	Vertical ramus elongation and mandibular advancement by endobuccal approach: Presentation of a new osteotomy technique	Excluded by title
568	Volumetric changes in the upper airway after bimaxillary surgery for skeletal class III malocclusions: a case series study using 3-dimensional cone-beam computed tomography	Excluded by title
569	What is the Risk of Developing Proximal Junctional Kyphosis During Growth Friendly Treatments for Early-onset Scoliosis?	Excluded by title
570	X-Ray Cephalometric Analysis of the Effects of Angle Class II and III Malocclusion on the Upper Airway Width and Hyoid Position between Parents and Children of Uygur Nationality	Excluded by title
571	Anterior open bite treated with a palatal crib and high-pull chin cup therapy. A prospective randomized study	Excluded by abstract
572	Effects of the reciprocal mini-chin cup appliance. The European Journal of Orthodontics	Excluded by abstract
573	Effects of treatment with a combined maxillary protraction and chincap appliance in skeletal Class III patients with different vertical skeletal morphologies	Excluded by abstract
574	Incremental effects of facemask therapy associated with intermaxillary mechanic	Excluded by abstract
575	Longitudinal growth changes in untreated subjects with Class II Division 1 malocclusion	Excluded by abstract
576	Long-term evaluation in Class II Division 1 patients after nonextraction orthodontic treatment – Dental cast analysis	Excluded by abstract
577	Nonextraction treatment of a skeletal Class III malocclusion. American journal of orthodontics and dentofacial orthopedics	Excluded by abstract
578	Upper molar distalization: Analysis of skeletal and dentoalveolar effects	Excluded by abstract
579	A Bayesian network meta-analysis of orthopaedic treatment in Class III malocclusion: Maxillary protraction with skeletal anchorage or a rapid maxillary expander	Excluded by abstract
580	A cephalometric and tomographic evaluation of Herbst treatment in the mixed dentition	Excluded by abstract
581	A cephalometric comparative study of the soft tissue airway dimensions in persons with hyperdivergent and normodivergent facial patterns	Excluded by abstract
582	A comparative cephalometric study of Class II, Division 1 nonextraction and extraction cases	Excluded by abstract
583	A comparison of the MARA and the AdvanSync functional appliances in the treatment of Class II malocclusion	Excluded by abstract
584	A nonsurgical approach to treatment of a high angle Class II, Division 1 malocclusion in a nongrowing patient	Excluded by abstract
585	A nonsurgical approach to treatment of high-angle Class II malocclusion	Excluded by abstract
586	A three-dimensional finite element analysis of molar distalization with a palatal plate, pendulum, and headgear according to molar eruption stage	Excluded by abstract
587	Activation-deactivation rapid palatal expansion and reverse headgear in Class III cases	Excluded by abstract
588	An American Board of Orhodontics case	Excluded by abstract
589	An evidence-based comparison of headgear and functional appliance therapy for the correction of Class II malocclusions	Excluded by abstract
590	Biomechanical basis of vertical dimension control during rapid palatal expansion therapy	Excluded by abstract
591	Cephalometric assessment of vertical control in the treatment of class II malocclusion with a combined maxillary splint	Excluded by abstract
592	Cephalometric changes after headgear anchored to the deciduous second molars in the early mixed dentition	Excluded by abstract
593	Cephalometric effects of the Jones Jig appliance followed by fixed appliances in Class II malocclusion treatment	Excluded by abstract
594	Cephalometric evaluation of the cervical spine posture following fixed functional therapy with Forsusâ„¢ appliance	Excluded by abstract
595	Cervical vertebral column morphology related to craniofacial morphology and head posture in preorthodontic children with Class II malocclusion and horizontal maxillary overjet	Excluded by abstract
596	Changes in the upper airway, hyoid bone and craniofacial morphology between patients treated with headgear activator and Herbst appliance: A retrospective study on lateral cephalometry	Excluded by abstract
597	Changes of hyoid, tongue and pharyngeal airway after mandibular setback surgery by intraoral vertical ramus osteotomy	Excluded by abstract
598	Changes of Occlusal Plane in Growing Patients With Increased Vertical Dimension During Class II Correction by Using Cervical Headgear	Excluded by abstract
599	Changes on facial profile in the mixed dentition, from natural growth and induced by Balters' bionator appliance	Excluded by abstract
600	Chin-throat anatomy: Normal relations and changes following orthognathic surgery and growth modification	Excluded by abstract
601	Class II correction in orthodontic patients utilizing the Mandibular Anterior Repositioning Appliance (MARA)	Excluded by abstract
602	Class II malocclusion correction: an American board of orthodontics ca	Excluded by abstract
603	CLASS-II - A COMPARISON OF ACTIVATOR AND ACTIVATOR HEADGEAR COMBINATION APPLIANCES	Excluded by abstract
604	Combination of bionator and high-pull headgear therapy in a skeletal open bite case	Excluded by abstract
605	Comparative efficiency of Class II malocclusion treatment with the pendulum appliance or two maxillary premolar extractions and edgewise appliances corrected]	Excluded by abstract
606	Comparative evaluation of maxillary protraction with or without skeletal anchorage	Excluded by abstract
607	Comparison of 2 modifications of the twin-block appliance in matched Class II samples	Excluded by abstract
608	Comparison of the short-term effects of facemask therapy preceded by conventional rapid maxillary expansion or by an alternate rapid maxillary expansions and constrictions protocol: A retrospective study	Excluded by abstract
609	Comparison of two maxillary protraction protocols: tooth-borne versus bone-anchored protraction facemask treatment	Excluded by abstract
610	Complications, impacts, and success rates of different approaches to treatment of Class II malocclusion in adolescents: A systematic review and meta-analysis	Excluded by abstract
611	Correction of a prominent premaxilla in a juvenile with a bilateral cleft lip and palate using a novel, hybrid function regulator, the FR-BCPPm, followed by preadjusted fixed appliances: A 20-year follow-up	Excluded by abstract
612	Craniofacial adaptations induced by chincup therapy in Class III patients	Excluded by abstract
613	Craniofacial displacement in response to varying headgear forces evaluated biomechanically with finite element analysis	Excluded by abstract
614	Craniofacial features of patients with Class III abnormalities: growth-related changes and effects of short-term and long-term chincup therapy	Excluded by abstract
615	Dental and skeletal effects of combined headgear used alone or in association with rapid maxillary expansion	Excluded by abstract
616	Dentofacial effects of skeletal anchored treatment modalities for the correction of maxillary retrognathia	Excluded by abstract
617	Differences between sliding mechanics with implant anchorage and straight-pull headgear and intermaxillary elastics in adults with bimaxillary protrusion	Excluded by abstract
618	Do functional orthodontic appliances stimulate mandibular growth in class II division 1 patients?	Excluded by abstract
619	Effects of a modified maxillary orthopaedic splint: a cephalometric evaluation	Excluded by abstract
620	Effects of activator and activator + anterior high-pull headgear on the growth direction of Class 2 ca	Excluded by abstract
621	Effects of orthodontic treatment on mandibular rotation and displacement in angle class II division I malocclusions	Excluded by abstract
622	Facilitating mandibular horizontal growth in a Class II high-angle case with a modified functional appliance	Excluded by abstract
623	Indication and effectiveness of the J-hook headgear]	Excluded by abstract
624	Is bodily advancement of the lower incisors possible?	Excluded by abstract
625	Long-term effect of the chincap on hard and soft tissues	Excluded by abstract
626	Long-term stability of orthodontic treatment and patient satisfaction - A systematic review	Excluded by abstract
627	Maurice Berman Prize 2003	Excluded by abstract
628	Maxillary traction splint: a cephalometric evaluation	Excluded by abstract
629	Monitoring growth during orthodontic treatment	Excluded by abstract
630	Nonsurgical correction of a skeletal Class II, Division 1, malocclusion with bilateral crossbite and anterior open bite	Excluded by abstract
631	Occlusal Plane and Skeletal Changes After Cervical Headgear Treatment With and Without Lower Utility Arch in Class II Growing Patients	Excluded by abstract
632	On the cephalometrics of skeletal change	Excluded by abstract
633	Overbite correction and sagittal changes: late mixed-dentition treatment effects	Excluded by abstract
634	Predictive value of masseter muscle thickness and bite force on Class II functional appliance treatment: a prospective controlled study	Excluded by abstract
635	Simultaneous distal movement of the maxillary first and second premolars can be achieved by distal movement of the maxillary first molar using a headgear	Excluded by abstract
636	Soft tissue changes following the treatment of Class II division 1 malocclusion using Headgear-activator appliance]	Excluded by abstract
637	The conversion of vertical growth to horizontal. A prospective pilot study of twelve consecutive patients treated by two different methods	Excluded by abstract
638	The effects of a modified protraction headgear on maxilla	Excluded by abstract

639	The effects of extraction and nonextraction treatment on the mandibular position	Excluded by abstract
640	Three-dimensional finite element analysis of maxillary protraction with labiolingual arches and implants	Excluded by abstract
641	Three-phase treatment concept for skeletal Class III growing patients with severe space deficiency: A report of three cases with skeletally anchored maxillary protraction	Excluded by abstract
642	Treatment and posttreatment effects of a facial mask combined with a bite-block appliance in Class III malocclusion	Excluded by abstract
643	Treatment effect of combined maxillary protraction and chincap appliance in severe skeletal Class III cases	Excluded by abstract
644	Treatment of a Class II, division 1 vertical growth pattern with severe anterior crowding	Excluded by abstract
645	Treatment of an Adult Patient with Skeletal Class II and Unilateral Cross-bite	Excluded by abstract
646	Treatment of Class II Div. I	Excluded by abstract
647	Treatment of Class II, Division 2 in the late growth period	Excluded by abstract
648	Treatment outcomes of growing Class II Division 1 patients with varying degrees of anteroposterior and vertical dysplasias, Part 1. Cephalometrics	Excluded by abstract
649	Tridimensional finite element analysis of teeth movement induced by different headgear forces	Excluded by abstract
650	Vertical control in fully-banded orthodontic treatment	Excluded by abstract
651	Effects of force magnitude on dental arches in cervical headgear therapy	Excluded; not relevant
652	Evaluation of the changes occurring in the vertical direction of the face in cases treated with activator + occipital headgear combination	No full text available
653	Effect of cervical headgear	Excluded; review
654	Effects of cervical headgear appliance: a systematic review	Excluded; review
655	Control of vertical dimension and chin position in class II malocclusion with miniscrew implants	Excluded; book chapter
656	An American Board of Orthodontics case report. A combined face mask-orthognathic surgical approach in the treatment of skeletal open bite and maxillary deficiency	Excluded; case report
657	An American Board of Orthodontics case report. Treatment of an open bite malocclusion	Excluded; case report
658	Armado como un rompecabezas: reporte de un caso clínico / Puzzle: case report	Excluded; case report
659	Case report KP. Treatment of a severe openbite excessive vertical pattern with an eclectic non-surgical approach	Excluded; case report
660	Case report: nonextraction treatment of a Class II division I malocclusion with procumbent anterior teeth	Excluded; case report
661	Case report: severe infraocclusion ankylosis occurring in siblings	Excluded; case report
662	Category 5: Class II Division 1 malocclusion with a high mandibular plane angle	Excluded; case report
663	Class II, Division 1 vertical pattern	Excluded; case report
664	Distalization with a C-DFD modified with mini-screws. A case report	Excluded; case report
665	Profile modifications induced by orthodontic forces studied by an analysis of the nasolabial angle Case report of maxillary protrusion]]	Excluded; case report
666	Severe dental Class II patient treated by activator followed by headgear therapy--a case report	Excluded; case report
667	Treatment of a patient with a mutilated Class II, Division 1 malocclusion and a dolichofacial skeletal pattern	Excluded; case report
668	Treatment of Class 2-high Angle cases using the combined activator-occipital headgear]	Excluded; case report
669	Use of onplants as stable anchorage for facemask treatment: a case report	Excluded; case report
670	Vertical control with a headgear-activator combination	Excluded; case report
671	Hans MG, Kishiyama C, Parker SH, Wolf GR, Noachtar R. Cephalometric evaluation of two treatment strategies for deep overbite correction. Angle Orthod. 1994;64(4):265-74; discussion 275-6.	Excluded; no control
672	Lima Filho RM, Lima AL, de Oliveira Ruellas AC. Longitudinal study of anteroposterior and vertical maxillary changes in skeletal class II patients treated with Kloehn cervical headgear. Angle Orthod. 2003 Apr;73(2):187-93.	Excluded; no control
673	Three-dimensional cone-beam computed technology evaluation of skeletal and dental changes in growing patients with Class II malocclusion treated with the cervical pull face-bow headgear appliance	Excluded; no control
674	Thurman MM, King GJ, Ramsay DS, Wheeler TT, Phillips C. The effect of an anterior biteplate on dental and skeletal Class II correction using headgears: a cephalometric study. Orthod Craniofac Res. 2011 Nov;14(4):213-21.	Excluded; no control
675	Treatment effects of the mandibular anterior repositioning appliance on patients with Class II malocclusion	Excluded; no control
676	Treatment effects on Class II division 1 high angle patients treated according to the Bioprogressive therapy (cervical headgear and lower utility arch)	Excluded; no control
677	Comparison of treatment outcomes between skeletal anchorage and extraoral anchorage in adults with maxillary dentoalveolar protrusion	Excluded; ineligible control
678	Dermaut LR, van den Eynde F, de Pauw G. Skeletal and dento-alveolar changes as a result of headgear activator therapy related to different vertical growth patterns. Eur J Orthod. 1992 Apr;14(2):140-6.	Excluded; co-intervention
679	Effects of activator and activator headgear treatment: comparison with untreated Class II subjects	Excluded; co-intervention
680	EVALUATION OF THE VERTICAL FORCES GENERATED BY THE CERVICAL BITEPLATE FACEBOW	Excluded; co-intervention
681	Thurman MM, King GJ, Ramsay DS, Wheeler TT, Phillips C. The effect of an anterior biteplate on dental and skeletal Class II correction using headgears: a cephalometric study. Orthodontics & Craniofacial Research. 2011 Nov;14(4):213-21.	Excluded; co-intervention
682	Vertical skeletal and dental changes in earlytreatment of class II malocclusion	Excluded; co-intervention
683	A tensor analysis to evaluate the effect of high-pull headgear on Class II malocclusions	Excluded; ineligible intervention
684	Baccetti T, Franchi L, Stahl F. Comparison of 2 comprehensive Class II treatment protocols including the bonded Herbst and headgear appliances: a double-blind study of consecutively treated patients at puberty. American Journal of Orthodontics and Dentofacial Orthopedics. 2009 Jun 1;135(6):698-e1.	Excluded; ineligible intervention
685	Class II: a comparison of activator and activator headgear combination appliances	Excluded; ineligible intervention
686	Comparative evaluation of a new removable Jasper Jumper functional appliance vs an activator-headgear combination	Excluded; ineligible intervention
687	Comparison of effects of cervical headgear treatment on skeletal facial changes when the treatment time is altered: a randomized controlled trial	Excluded; ineligible intervention
688	Comparison of modified Teuscher and van Beek functional appliance therapies in high-angle cases	Excluded; ineligible intervention
689	Effectiveness of twin blocks and extraoral maxillary splint (Thurow) appliances for the correction of Class II relationships	Excluded; ineligible intervention
690	Effects of activator and high-pull headgear combination therapy	Excluded; ineligible intervention
691	Effects of activator and high-pull headgear combination therapy: skeletal, dentoalveolar, and soft tissue profile changes	Excluded; ineligible intervention
692	Heavy intermittent cervical traction in class II treatment: a longitudinal cephalometric assessment	Excluded; ineligible intervention
693	Holographic and cephalometric study of the relationship between craniofacial morphology and the initial reactions to high-pull headgear traction	Excluded; ineligible intervention
694	Maxillary "en masse" high-pull traction in Class II division 1 subjects: Which kind of skeletal outcomes does it produce?	Excluded; ineligible intervention
695	Skeletal and dental components of Class II correction with the bionator and removable headgear splint appliances	Excluded; ineligible intervention
696	Skeletal and dento-alveolar changes as a result of headgear activator therapy related to different vertical growth patterns	Excluded; ineligible intervention
697	Treatment with an orthopedic appliance system in relation to treatment intensity and growth periods. A study of initial effects	Excluded; ineligible intervention
698	Efficiency in the correction of Class II division 1 malocclusions with prominent upper incisors and increased overjet: a comparison between two methods	Excluded; intervention unclear
699	effects of cervical headgear treatment on skeletal facial changes when the treatment time is altered	Excluded; ineligible outcome
700	Boecler PR, Riolo ML, Keeling SD, TenHave TR. Skeletal changes associated with extraoral appliance therapy: an evaluation of 200 consecutively treated cases. The Angle Orthodontist. 1989 Dec;59(4):264-70.	Excluded; ineligible outcome
701	Changes in upper airway width associated with Class II treatments (headgear vs activator) and different growth patterns	Excluded; ineligible outcome
702	Comparison of 2 comprehensive Class II treatment protocols including the bonded Herbst and headgear appliances: a double-blind study of consecutively treated patients at puberty	Excluded; ineligible outcome
703	Control of vertical dimension in the treatment of Class II malocclusion using a combined activator and extraoral traction appliance]	Excluded; ineligible outcome
704	Early headgear effects on the eruption pattern of the maxillary canines	Excluded; ineligible outcome
705	Effect of maxillary anteroposterior position on profile esthetics in headgear-treated patients	Excluded; ineligible outcome
706	Julku J, Pirilä-Parkkinen K, Tolvanen M, Pirttiniemi P. Comparison of effects of cervical headgear treatment on skeletal facial changes when the treatment time is altered: a randomized controlled trial. European journal of orthodontics. 2019 Nov 15;41(6):631-40.	Excluded; ineligible outcome
707	Long-term soft-tissue response to orthodontic treatment with early cervical headgear--a randomized study	Excluded; ineligible outcome
708	Orthopedic cervical headgear with an expanded inner bow in class II correction	Excluded; ineligible outcome
709	Polat-Ozsoy O, Gokcelik A, Güngör-Acar A, Kircelli BH. Soft tissue profile after distal molar movement with a pendulum K-loop appliance versus cervical headgear. The Angle Orthodontist. 2008 Mar;78(2):317-23.	Excluded; ineligible outcome
710	Skeletal changes associated with plate-headgear therapy in the early mixed dentition	Excluded; ineligible outcome
711	Talvitie T, Helminen M, Karsila S, Varho R, Signorelli L, Pirttiniemi P, Peltomäki T. The impact of force magnitude on the first and second maxillary molars in cervical headgear therapy. European Journal of Orthodontics. 2021 Dec;43(6):648-57.	Excluded; ineligible outcome
712	The impact of force magnitude on the first and second maxillary molars in cervical headgear therapy	Excluded; ineligible outcome
713	Treatment effects of headgear-Herbst appliance]	Excluded; ineligible outcome

714	TREATMENT OF CLASS-II HIGH ANGLE MALOCCLUSIONS WITH THE HERBST APPLIANCE - A CEPHALOMETRIC INVESTIGATION	Excluded; ineligible outcome
715	Treatment timing for an orthopedic approach to patients with increased vertical dimension	Excluded; ineligible outcome
716	Early or late cervical traction therapy of Class II malocclusion in the mixed dentition	Excluded; outcome unclear
717	Hans MG, Kishiyama C, Parker SH, Wolf GR, Noachtar R. Cephalometric evaluation of two treatment strategies for deep overbite correction. Angle Orthod. 1994;64(4):265-74; discussion 275-6.	Excluded; no Class II
718	Alió-Sanz J, Iglesias-Conde C, Lorenzo-Pernía J, Iglesias-Linares A, Mendoza-Mendoza A, Solano-Reina E. Effects on the maxilla and cranial base caused by cervical headgear: a longitudinal study. Med Oral Patol Oral Cir Bucal. 2012 Sep 1;17(5):e845-51.	Included
719	Antonarakis GS, Kiliaridis S. Treating Class II malocclusion in children. Vertical skeletal effects of high-pull or low-pull headgear during comprehensive orthodontic treatment and retention. Orthod Craniofac Res. 2015 May;18(2):86-95.	Included
720	Bondemark L, Karlsson I. Extraoral vs intraoral appliance for distal movement of maxillary first molars: a randomized controlled trial. The Angle Orthodontist. 2005 Sep;75(5):699-706.	Included
721	Burke M, Jacobson A. Vertical changes in high-angle Class II, division 1 patients treated with cervical or occipital pull headgear. Am J Orthod Dentofacial Orthop. 1992 Dec;102(6):501-8.	Included
722	Cook AH, Sellke TA, BeGole EA. Control of the vertical dimension in Class II correction using a cervical headgear and lower utility arch in growing patients. Part I. Am J Orthod Dentofacial Orthop. 1994 Oct;106(4):376-88. doi: 10.1016/S0889-5406(94)70059-1. Erratum in: Am J Orthod Dentofacial Orthop 1995 Mar;107(3):308.	Included
723	Derringer K. A cephalometric study to compare the effects of cervical traction and Andresen therapy in the treatment of Class II division 1 malocclusion. Part 1--Skeletal changes. Br J Orthod. 1990 Feb;17(1):33-46.	Included
724	Freitas MR, Lima DV, Freitas KM, Janson G, Henriques JF. Cephalometric evaluation of Class II malocclusion treatment with cervical headgear and mandibular fixed appliances. Eur J Orthod. 2008 Oct;30(5):477-82.	Included
725	Gkantidis N, Halazonetis DJ, Alexandropoulos E, Haralabakis NB. Treatment strategies for patients with hyperdivergent Class II Division 1 malocclusion: is vertical dimension affected? Am J Orthod Dentofacial Orthop. 2011 Sep;140(3):346-55.	Included
726	Haralabakis NB, Sifakakis IB. The effect of cervical headgear on patients with high or low mandibular plane angles and the "myth" of posterior mandibular rotation. Am J Orthod Dentofacial Orthop. 2004 Sep;126(3):310-7.	Included
727	Kim KR, Muhl ZF. Changes in mandibular growth direction during and after cervical headgear treatment. Am J Orthod Dentofacial Orthop. 2001 May;119(5):522-30.	Included
728	Lione R, Franchi L, Laganà G, Cozza P. Effects of cervical headgear and pendulum appliance on vertical dimension in growing subjects: a retrospective controlled clinical trial. Eur J Orthod. 2015 Jun;37(3):338-44.	Included
729	Mäntysaari R, Kantomaa T, Pirttiniemi P, Pykäläinen A. The effects of early headgear treatment on dental arches and craniofacial morphology: a report of a 2 year randomized study. Eur J Orthod. 2004 Feb;26(1):59-64.	Included
730	Mossaz CF, Byloff FK, Kiliaridis S. Cervical headgear vs pendulum appliance for the treatment of moderate skeletal Class II malocclusion. Am J Orthod Dentofacial Orthop. 2007 Nov;132(5):616-23.	Included
731	Park CO, Sa'aed NL, Bayome M, Park JH, Kook YA, Park YS, Han SH. Comparison of treatment effects between the modified C-palatal plate and cervical pull headgear for total arch distalization in adults. Korean J Orthod. 2017 Nov;47(6):375-383.	Included
732	Rosa AJ, Nascimento RRD, Mucha JN, Vilella OV. Effects of the cervical headgear in growing Angle Class II malocclusion patients: a prospective study. Dental Press J Orthod. 2020 Mar;25(2):25-31.	Included
733	Sambataro S, Fastuca R, Oppermann NJ, Lorusso P, Baccetti T, Franchi L, Caprioglio A. Cephalometric changes in growing patients with increased vertical dimension treated with cervical headgear. J Orofac Orthop. 2017 Jul;78(4):312-320.	Included
734	Sambataro S, Lorusso P, Caprioglio A, Franchi L, Cicciù M, Fastuca R. Changes of Occlusal Plane in Growing Patients With Increased Vertical Dimension During Class II Correction by Using Cervical Headgear. J Craniofac Surg. 2020 Jan/Feb;31(1):172-177.	Included
735	Sambataro S, Bocchieri S, Fastuca R, Giuntini V, Fiorillo L, Cicciù M, Caprioglio A. Occlusal Plane and Skeletal Changes After Cervical Headgear Treatment With and Without Lower Utility Arch in Class II Growing Patients. J Craniofac Surg. 2021 May 1;32(3):1152-1156.	Included
736	Sambataro S, Rossi O, Bocchieri S, Fastuca R, Oppermann N, Levrini L, Cicciù M, Caprioglio A. Comparison of cephalometric changes in Class II growing patients with increased vertical dimension after high-pull and cervical headgear treatment. Eur J Paediatr Dent. 2023 Feb;24(1):36-41.	Included
737	Ulger G, Arun T, Sayinsu K, Isik F. The role of cervical headgear and lower utility arch in the control of the vertical dimension. Am J Orthod Dentofacial Orthop. 2006 Oct;130(4):492-501.	Included
738	Zervas ED, Galang-Boquiren MT, Obrez A, Costa Viana MG, Oppermann N, Sanchez F, Romero EG, Kusnoto B. Change in the vertical dimension of Class II Division 1 patients after use of cervical or high-pull headgear. Am J Orthod Dentofacial Orthop. 2016 Nov;150(5):771-781.	Included

Supplementary Table 3. Assessment of included randomized studies with the ROB-2 tool

Study	Randomization process	Deviations from intended interventions	Missing outcome data	Measurement of the outcome	Selection of the reported result	Overall Bias
Bondermark 2005	High	High	Low	High	Some concerns	High
Mantysaari 2004	some concern	High	Low	High	Some concerns	High

Supplementary Table 4. Assessment of included randomized studies with the ROB-2 tool

Domain	Reference	Ulger 2006	Sambataro 2017coll	Cook 1994	Rosa 2020	Mossaz 2005	Gkantidis 2011	Antonarakis 2014	Zervas 2016	Burke 1992	Park 2017	Kim 2000	Lione 2014	Freitas 2008	Aliò-Sanz 2012	Haralabakis 2003	Derringer 1990
**Confounding**	**1.1**	Y	Y	Y	Y	Y	Y	Y	Y	Y	Y	Y	Y	Y	Y	Y	Y
	**1.2**	N	N	N	N	N	Y	N	N	Y	N	Y	N	N	N	N	N
	**1.3**	N	N	N	N	N	N	N	N	N	N	N	N	N	N	N	N
	**1.4**	Y	NI	PY	PY	Y	NI	Y	NI	NI	PN	y	NI	NI	Y	PY	Y
	**1.5**	Y	NA	Y	Y	Y	NA	Y	NA	NA	NA	y	NA	NA	y	y	y
	**1.6**	PN	PN	PN	PN	PN	PN	PN	PN	PN	PN	PN	PN	PN	PN	PN	PN
	**1.7**	N	N	N	N	N	N	N	N	N	N	N	N	N	NA	N	N
	**1.8**	NA	NA	NA	NA	NA	NA	NA	NA	NA	NA	NA	NA	NA	NA	NA	NA
	**Judgment**	(M)	(S)	(M)	(M)	(M)	(S)	(S)	(S)	(S)	(S)	(S)	(S)	(S)	(M)	(M)	(M)
**Selection of participants into study**	**2.1**	N	N	N	N	PN	N	Y	N	N	N	N	N	N	N	N	PN
	**2.2**	NA	NA	NA	NA	NA	NA	Y	NA	NA	NA	NA	NA	NA	NA	NA	NA
	**2.3**	NA	NA	NA	NA	NA	NA	Y	NA	NA	NA	NA	NA	NA	NA	NA	NA
	**2.4**	Y	Y	Y	Y	PY	N	Y	Y	NI	Y	Y	Y	Y	PY	Y	Y
	**2.5**	NA	NA	NA	NA	NA	N	NA	NA	NA	NA	NA	NA	NA	NA	NA	NA
	**Judgment**	(L)	(L)	(L)	(L)	(L)	(S)	(S)	(L)	(L)	(L)	(L)	(L)	(L)	(M)	(L)	(L)
**Classification of intervention**	**3.1**	Y	Y	Y	Y	Y	Y	Y	Y	Y	Y	Y	Y	Y	Y	Y	Y
	**3.2**	Y	Y	Y	Y	Y	Y	Y	Y	Y	Y	Y	Y	Y	Y	Y	Y
	**3.3**	N	N	N	N	N	N	N	N	N	N	N	N	N	N	N	N
	**Judgment**	(L)	(L)	(L)	(L)	(L)	(L)	(L)	(L)	(L)	(L)	(L)	(L)	(L)	(L)	(L)	(L)
**Deviations from intended interventions**	**4.1**	N	N	PN	N	N	NI	NI	PY	NI	PY	N	N	N	N	NI	NI
	**4.2**	NA	NA	NA	NA	NA	NA	NA	NI	NA	NI	NA	NA	NA	NA	NA	NA
	**4.3**	Y	Y	Y	Y	PY	N	PY	Y	Y	PY	N	PN	N	Y	PY	Y
	**4.4**	Y	Y	PY	Y	Y	NI	NI	PN	NI	PN	Y	Y	Y	Y	NI	NI
	**4.5**	PY	PY	PY	PY	PY	PY	PY	PY	PY	PY	PY	PY	PY	PY	PY	PY
	**4.6**	NA	NA	NA	NA	NA	NA	NA	NA	NA	NA	NA	NA	NA	NA	NA	NA
	**Judgment**	(L)	(L)	(L)	(L)	(L)	(M)	(S)	(M)	(L)	(S)	(M)	(M)	(M)	low	low	low
**Missing data**	**5.1**	Y	Y	NI	Y	Y	Y	NI	Y	NI	NI	Y	Y	Y	NI	NI	Y
	**5.2**	N	N	PN	N	N	N	PN	N	PN	PN	N	N	N	PN	PN	N
	**5.3**	N	N	PN	N	N	N	PN	N	PN	PN	N	N	N	PN	PN	N
	**5.4**	NA	NA	NA	NA	NA	NA	NA	NA	NA	NA	NA	NA	NA	NA	NA	NA
	**5.5**	NA	NA	NA	NA	NA	NA	NA	NA	NA	NA	NA	NA	NA	NA	NA	NA
	**Judgment**	(L)	(L)	NI	(L)	(L)	(L)	NI	(L)	NI	NI	low	low	low	NI	NI	low
**Measurement of Outcomes**	**6.1**	PN	PN	PN	PN	PN	PN	PN	PN	PN	PN	PN	PN	PN	PN	PN	PN
	**6.2**	PY	PY	PY	PY	PY	PY	PY	PY	PY	PY	PY	PY	PY	PY	PY	PY
	**6.3**	PY	PY	PY	PY	PY	PY	PY	PY	PY	PY	PY	PY	PY	PY	PY	PY
	**6.4**	PN	PN	PN	PN	PN	PN	PN	PN	PN	PN	PN	PN	PN	PN	PN	PN
	**Judgment**	(M)	(M)	(M)	(M)	(M)	(M)	(M)	(M)	(M)	(M)	(M)	(M)	(M)	(M)	(M)	(M)
**Selection of reported results**	**7.1**	N	PN	PN	PN	PN	PN	PN	PN	PN	PN	PN	PN	PN	PN	PN	PN
	**7.2**	PN	PN	PN	PN	PN	PN	PN	PN	PN	PN	PN	PN	PN	PN	PN	PN
	**7.3**	PN	PN	PN	PN	PN	PN	PN	PN	PN	PN	PN	PN	PN	PN	PN	PN
	**Judgment**	low	low	low	low	low	low	low	low	low	low	low	low	low	low	low	low
**Overall**	**Judgment**	(M)	(S)	(M)	(M)	(M)	(S)	(S)	(S)	(S)	(S)	(M)	(S)	(S)	(M)	(M)	(M)

(L), low; (M), moderate; (S), serious.

Supplementary Table 5. Results of single studies comparing cervical headgear with other alternatives.

Nr	Comparison	Outcome	MD (95% CI)	P	Clinically relevant
1	cHG vs control	NL-ML	0.20 (-0.58, 0.98)	0.62	-
2	cHG vs hp-HG	SN-NL	-0.30 (-1.65, 1.05)	0.66	-
3		FH-NL	2.24 (1.14, 3.34)	<0.001	No
4		FH-ML	-1.04 (-2.14, 0.06)	0.06	-
5		NL-ML	-2.41 (-4.64, -0.18)	0.04	No
6		ArGo	1.58 (-0.11, 3.27)	0.07	-
7	cHG vs cHG+LUA	SN-ML	0.08 (-0.61, 0.77)	0.82	-
8		FH-NL	-0.36 (-1.18, 0.46)	0.39	-
9		FH-ML	0.35 (-0.36, 1.06)	0.33	-
10		ArGo	0.75 (-0.25, 1.75)	0.14	-
11		SGo	-0.50 (-1.50, 0.50)	0.33	-
12	cHG vs intraoral distalizer	SN-NL	0.80 (-1.50, 0.50)	0.27	-
13		FH-NL	-0.46 (-1.42, 0.50)	0.35	-
14	cHG vs functional appliance	SN-NL	0.14 (-0.62, 0.90)	0.72	-
15		NSGn	0.61 (-0.62, 0.90)	0.06	-
16		ArGo	0.01 (-1.44, 1.46)	0.99	-
17	cHG vs intrusive mechanics	SN-ML	0.29 (-0.83, 1.41)	0.61	-
18		FH-ML	0.25 (-1.04, 1.54)	0.71	-

cHG, cervical headgear; CI, confidence interval; hp-HG, high-pull headgear; LUA, lower utility arch; MD, mean difference.

Supplementary Table 6. Meta-regression analyses for meta-analyses with at least 5 included studies comparing cervical headgear to untreated controls.

	Mean age			% male in sample	P		Mean duration	P
**Outcome**	**Coefficient (95% CI)**	**P**		**Coefficient (95% CI)**	**P**		**Coefficient (95% CI)**	**P**
SN-ML / FH-ML	0.01 (-0.21, 0.22)	0.90		-0.03 (-0.05, -0.01)	0.01		0 (-0.01, 0.02)	0.66
SN-NL / FH-NL	-0.22 (-0.84, 0.40)	0.48		-0.09 (-0.24, 0.06)	0.26		-0.03 (-0.09, 0.04)	0.45

CI, confidence interval; RCT, randomised clinical trial; SMD, standardized mean difference.

Supplementary Table 7. Sensitivity analyses for meta-analyses with at least 5 included studies comparing cervical headgear to untreated controls.

	RCT vs non-RCT			Prospective vs retrospective			Large vs small		
	RCT	Non-RCT		Prospective	Retrospective		Large	Small	
**Outcome**	**n<br>SMD<br>(95% CI)**	**n<br>SMD<br>(95% CI)**	**P**	**n<br>SMD<br>(95% CI)**	**n<br>SMD<br>(95% CI)**	**P**	**n<br>SMD<br>(95% CI)**	**n<br>SMD<br>(95% CI)**	**P**
SN-ML / FH-ML	n=0	n=9	-	n=1<br>0.05<br>(-0.54, 0.63)	n=7<br>0.24<br>(-0.08, 0.56)	0.05	n=4<br>0.20<br>(-0.50, 0.90)	n=4<br>0.24<br>(-0.08, 0.55)	0.88
SN-NL / FH-NL	n=1<br>0.61<br>(0.09, 1.13)	n=8<br>0.87<br>(-0.59, 2.33)	0.68	n=1<br>0.61<br>(0.09, 1.13)	n=8<br>0.87<br>(-0.59, 2.33)	0.68	n=2<br>2.15<br>(-21.13, 25.43)	n=5<br>0.40<br>(0.06, 0.74)	0.34

CI, confidence interval; RCT, randomised clinical trial; SMD, standardized mean difference.

Supplementary Figure 1. Risk of bias of included non-randomized studies with the ROBINS-I tool.

Study	Experimental	Comparator	Outcome	D1	D2	D3	D4	D5	Overall
Bondermark 2005	CPH	Nance TPA	SNMPA	-	-	+	-	!	-
Mantysaari 2004	CPH	No Treatment	SN-PP, MMA	!	-	+	-	!	-

+ Low risk

! Some concerns

- High risk

Supplementary Figure 2. Risk of bias of included non-randomized studies with the ROBINS-I tool.

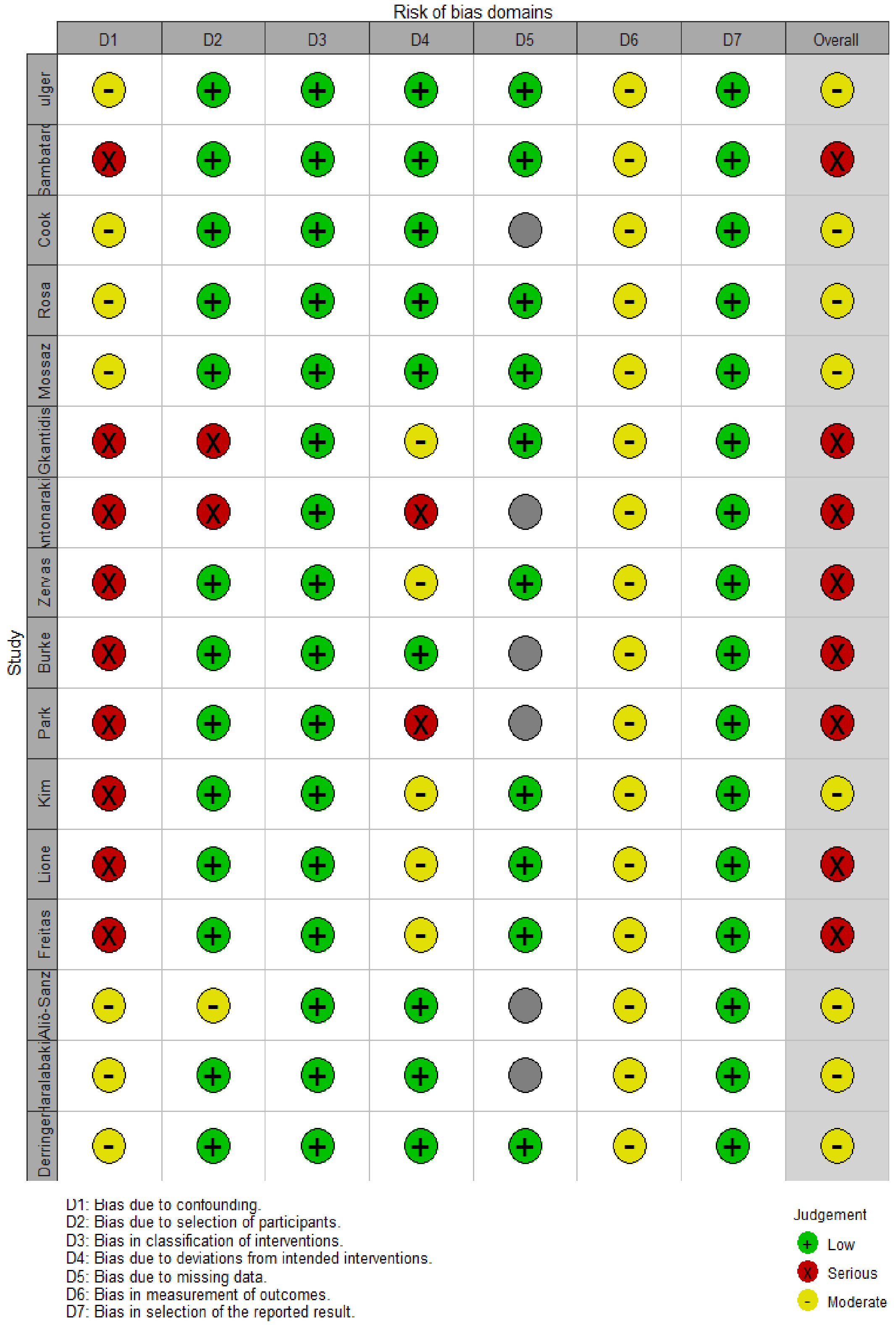

Supplementary Figure 3. Summary bar plot for ROBINS-I of the included non-randomized studies

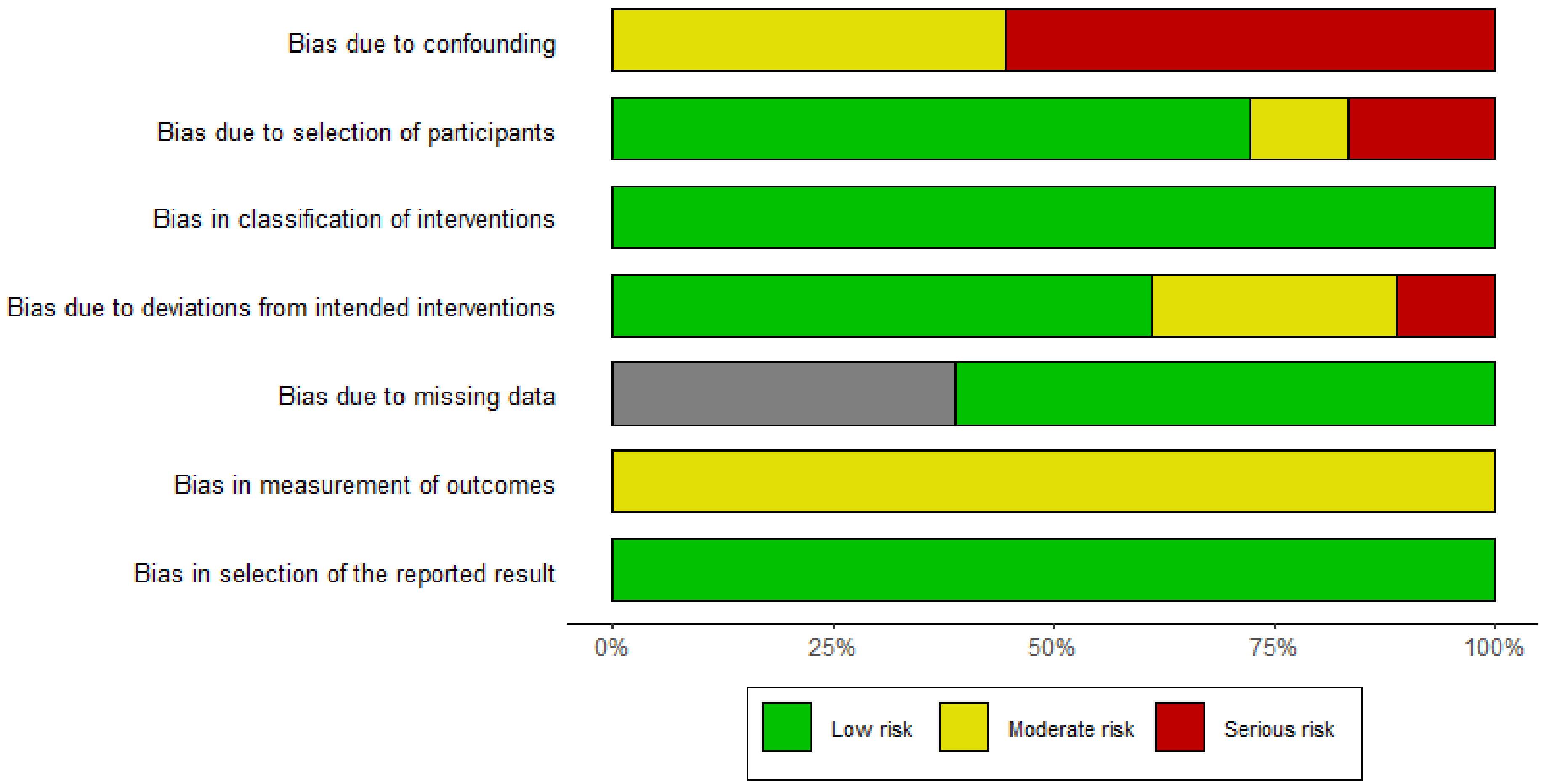

Supplementary Figure 4. Forest plot for the comparison of cervical headgear versus control (no treatment); outcome: SN-NL / FH-NL.

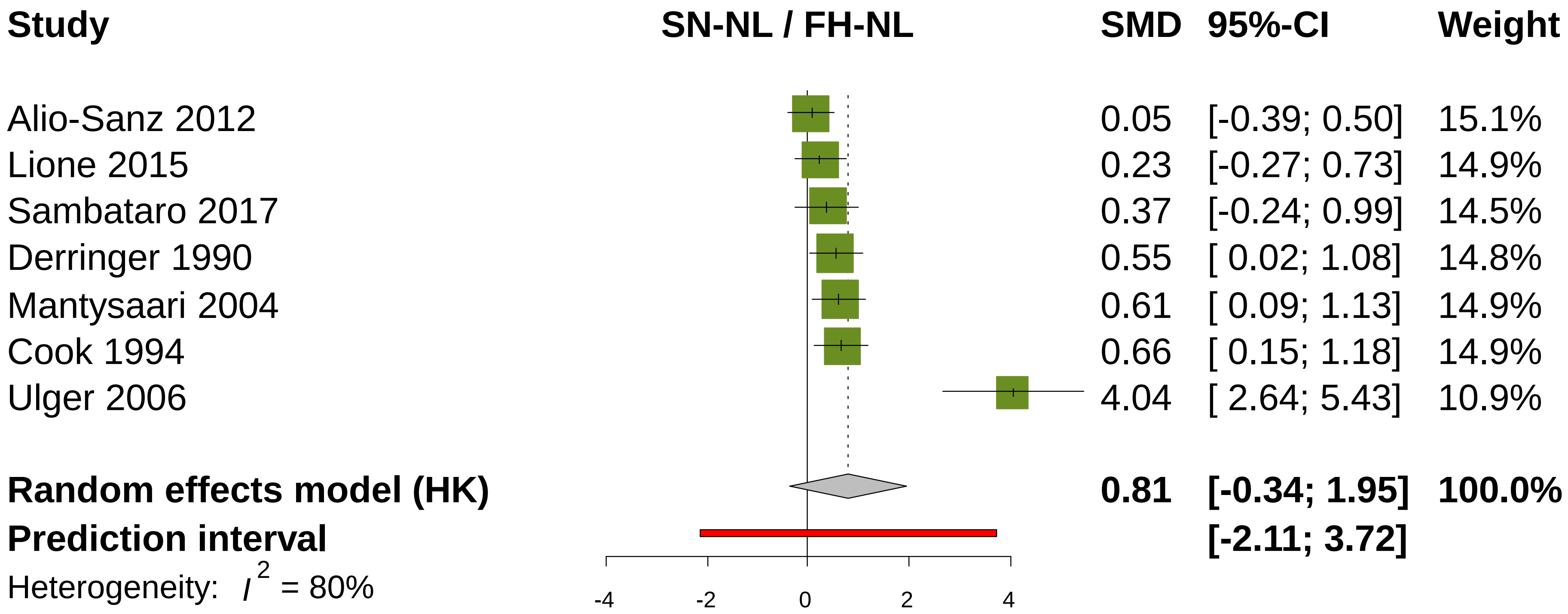

CI, confidence interval; SMD, standarised mean difference.

Supplementary Figure 5. Forest plot for the comparison of cervical headgear versus control (no treatment); outcome: N-S-Gn.

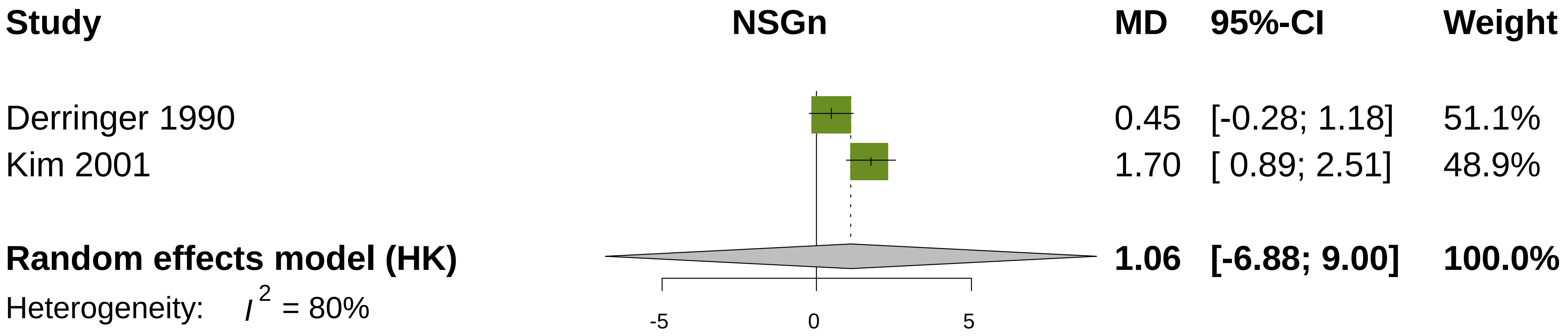

CI, confidence interval; MD, mean difference.

Supplementary Figure 6. Forest plot for the comparison of cervical headgear versus control (no treatment); outcome: BaN-PtGn.

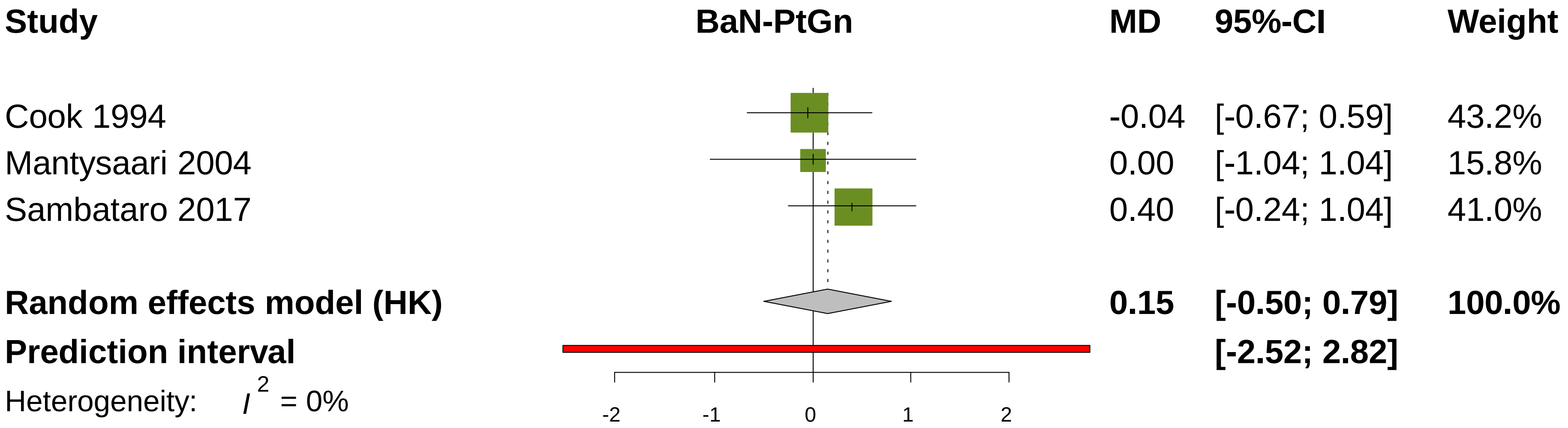

CI, confidence interval; MD, mean difference.

Supplementary Figure 7. Forest plot for the comparison of cervical headgear versus control (no treatment); outcome: Ar-Go.

Study	MD	95%-CI	Weight
Kim 2001	0.20	[-1.63; 2.03]	30.0%
Derringer 1990	2.79	[1.19; 4.39]	32.0%
Ulger 2006	3.58	[2.76; 4.40]	38.1%
**Random effects model (HK)**	**2.31**	**[-2.01; 6.64]**	**100.0%**
**Prediction interval**		**[-21.18; 25.81]**	

Heterogeneity: $I^2 = 82\%$

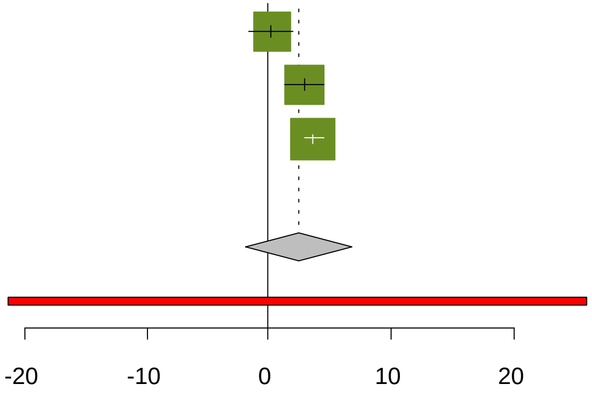

CI, confidence interval; MD, mean difference.

Supplementary Figure 8. Forest plot for the comparison of cervical headgear high-pull headgear; outcome: SN-ML / FH-ML.

Study	SN-ML / FH-ML	SMD	95%-CI	Weight
Zervas 2016		-0.57	[-1.20; 0.05]	23.9%
Sambataro 2017col		-0.13	[-0.80; 0.54]	22.6%
Antonarakis 2015		-0.03	[-0.54; 0.47]	27.7%
Burke 1992		0.62	[0.06; 1.18]	25.9%
**Random effects model (HK)**		**-0.01**	**[-0.80; 0.77]**	**100.0%**
**Prediction interval**			**[-2.00; 1.97]**	
Heterogeneity: I^2= 63%				

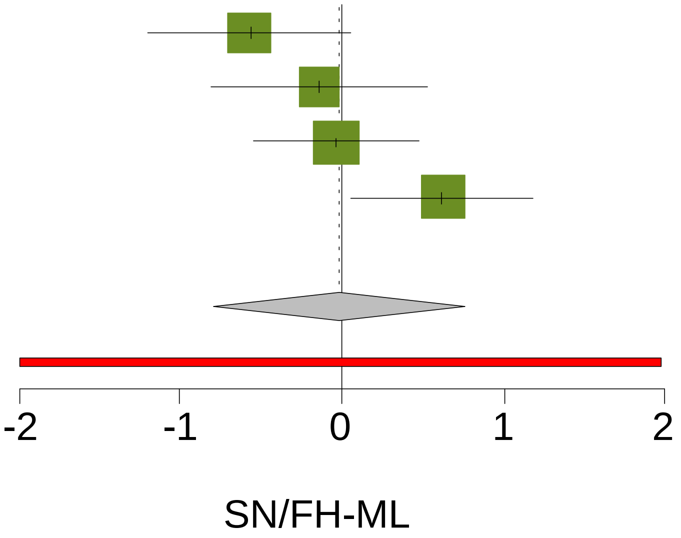

CI, confidence interval; SMD, standarised mean difference.

Supplementary Figure 9. Forest plot for the comparison of cervical headgear high-pull headgear; outcome: SN-NL / FH-NL.

Study	SMD	95%-CI	Weight
Antonarakis 2015	-0.11	[-0.62; 0.40]	35.9%
Sambataro 2017col	0.49	[-0.19; 1.17]	31.9%
Zervas 2016	1.21	[0.54; 1.88]	32.2%
**Random effects model (HK)**	**0.51**	**[-1.15; 2.16]**	**100.0%**
**Prediction interval**		**[-8.51; 9.52]**	
Heterogeneity: $I^2 = 79\%$			

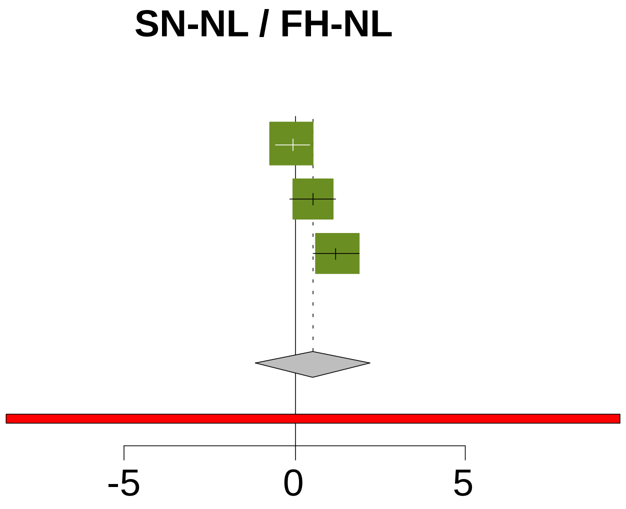

CI, confidence interval; SMD, standarised mean difference.

Supplementary Figure 10. Forest plot for the comparison of cervical headgear high-pull headgear; outcome: BaN-PtGn.

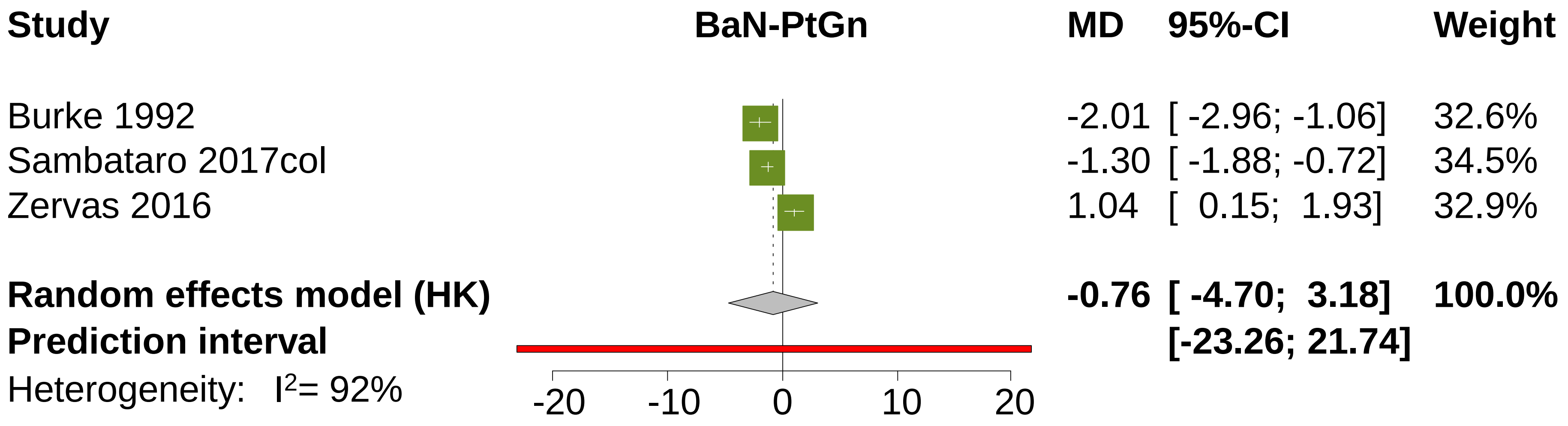

CI, confidence interval; MD, mean difference.

Supplementary Figure 11. Forest plot for the comparison of cervical headgear versus cervical headgear plus lower utility arch; outcome: SN-ML / FH-ML.

Study	SMD	95%-CI	Weight
Sambataro 2017col	-0.17	[-0.80; 0.46]	31.7%
Ulger 2006	0.09	[-0.71; 0.89]	19.6%
Cook 1994	0.25	[-0.26; 0.75]	48.7%
**Random effects model (HK)**	**0.08**	**[-0.47; 0.64]**	**100.0%**
**Prediction interval**		**[-2.21; 2.38]**	
Heterogeneity: $I^2 = 0\%$			

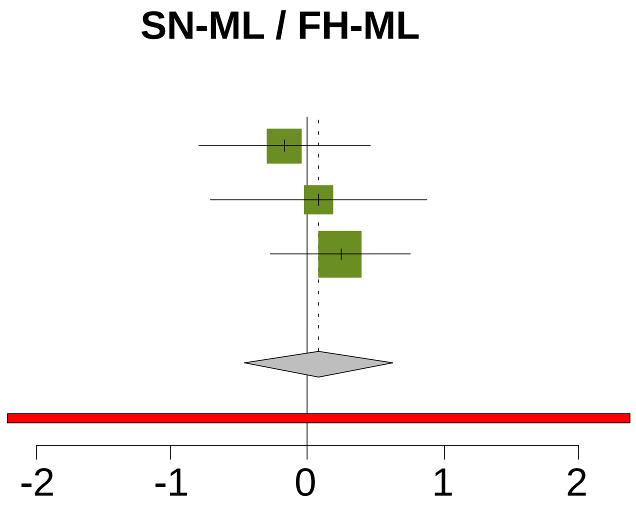

CI, confidence interval; SMD, standarised mean difference.

Supplementary Figure 12. Forest plot for the comparison of cervical headgear versus cervical headgear plus lower utility arch; outcome: SN-NL.

Study	SN-NL / FH-NL	SMD	95%-CI	Weight
Ulger 2006		-0.20	[-1.00; 0.60]	19.7%
Sambataro 2017col		0.05	[-0.57; 0.68]	32.1%
Cook 1994		0.42	[-0.09; 0.93]	48.2%
**Random effects model (HK)**		**0.18**	**[-0.57; 0.94]**	**100.0%**
**Prediction interval**			**[-2.14; 2.50]**	
Heterogeneity: $I^2 = 0\%$				

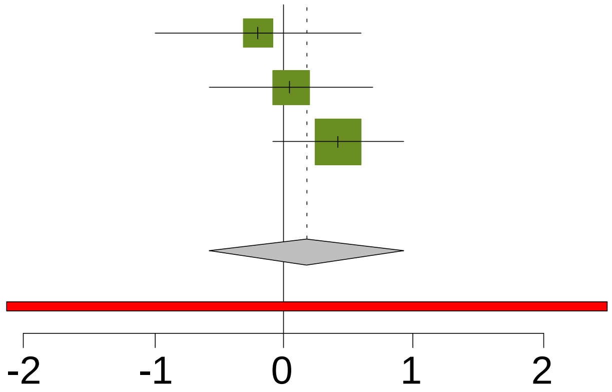

CI, confidence interval; SMD, standardized mean difference.

Supplementary Figure 13. Forest plot for the comparison of cervical headgear versus intraoral distaliser; outcome: SN-ML.

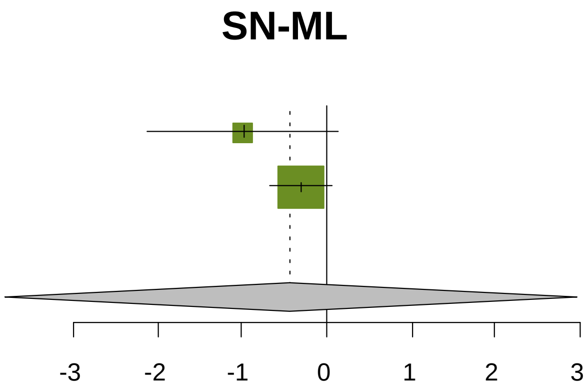

Study	MD	95%-CI	Weight
Mossaz 2007	-0.99	[-2.12; 0.14]	18.5%
Bondemark 2005	-0.30	[-0.66; 0.06]	81.5%
**Random effects model (HK)**	**-0.43**	**[-3.83; 2.97]**	**100.0%**
Heterogeneity: $I^2 = 22\%$			

CI, confidence interval; MD, mean difference.

Supplementary Figure 14. Forest plot for the comparison of cervical headgear versus intraoral distaliser; outcome: SN-NL / FH-NL.

Study	SMD	95%-CI	Weight
Park 2017	-0.28	[-0.87; 0.32]	42.4%
Lione 2015	0.25	[-0.19; 0.69]	57.6%
**Random effects model (HK)**	**0.02**	**[-3.26; 3.31]**	**100.0%**
Heterogeneity: $I^2 = 48\%$			

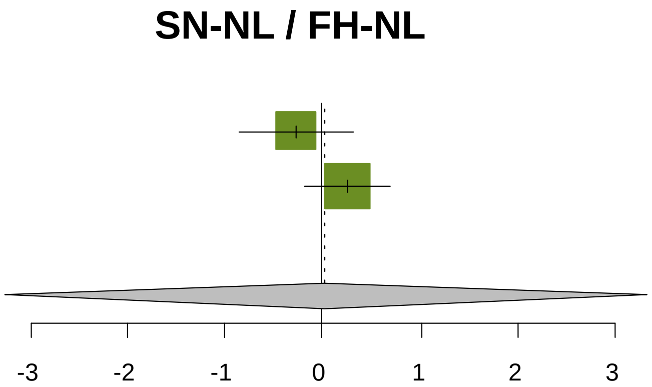

CI, confidence interval; SMD, standarised mean difference.

Supplementary Figure 15. Forest plot for the comparison of cervical headgear versus functional appliance; outcome: SN-ML / FH-ML.

Study	SN-ML	MD	95%-CI	Weight
Haralabakis 2003		-0.50	[-1.76; 0.76]	40.0%
Derringer 1990		0.20	[-0.83; 1.23]	60.0%
**Random effects model (HK)**		**-0.08**	**[-4.44; 4.28]**	**100.0%**
Heterogeneity: $I^2 = 0\%$				

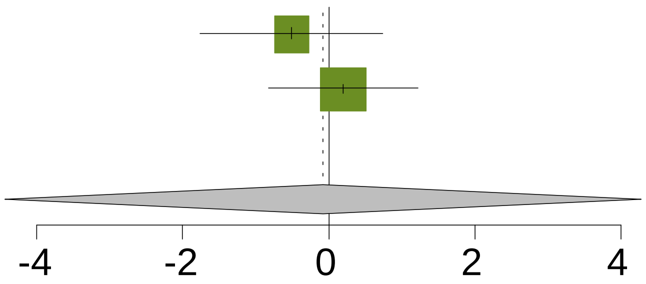

CI, confidence interval; MD, mean difference.
